# The Biological Role and Therapeutic Potential of NK Cells in Hematological and Solid Tumors

**DOI:** 10.3390/ijms222111385

**Published:** 2021-10-21

**Authors:** Rodion A. Velichinskii, Maria A. Streltsova, Sofya A. Kust, Alexander M. Sapozhnikov, Elena I. Kovalenko

**Affiliations:** Laboratory of Cell Interactions, Immunology Department, Shemyakin and Ovchinnikov Institute of Bioorganic Chemistry, Russian Academy of Sciences, ul. Miklukho-Maklaya, 117997 Moscow, Russia; rodicvelic@gmail.com (R.A.V.); mstreltsova@mail.ru (M.A.S.); sonya.erokhina@gmail.com (S.A.K.); amsap@mail.ru (A.M.S.)

**Keywords:** NK cells, hematological tumors, hematological tumors, NK cell immunotherapy, genetic modification

## Abstract

NK cells are an attractive target for cancer immunotherapy due to their potent antitumor activity. The main advantage of using NK cells as cytotoxic effectors over T cells is a reduced risk of graft versus host disease. At present, several variants of NK-cell-based therapies are undergoing clinical trials and show considerable effectiveness for hematological tumors. In these types of cancers, the immune cells themselves often undergo malignant transformation, which determines the features of the disease. In contrast, the current use of NK cells as therapeutic agents for the treatment of solid tumors is much less promising. Most studies are at the stage of preclinical investigation, but few progress to clinical trials. Low efficiency of NK cell migration and functional activity in the tumor environment are currently considered the major barriers to NK cell anti-tumor therapies. Various therapeutic combinations, genetic engineering methods, alternative sources for obtaining NK cells, and other techniques are aiming at the development of promising NK cell anticancer therapies, regardless of tumorigenesis. In this review, we compare the role of NK cells in the pathogenesis of hematological and solid tumors and discuss current prospects of NK-cell-based therapy for hematological and solid tumors.

## 1. Introduction

**Figure 1:**
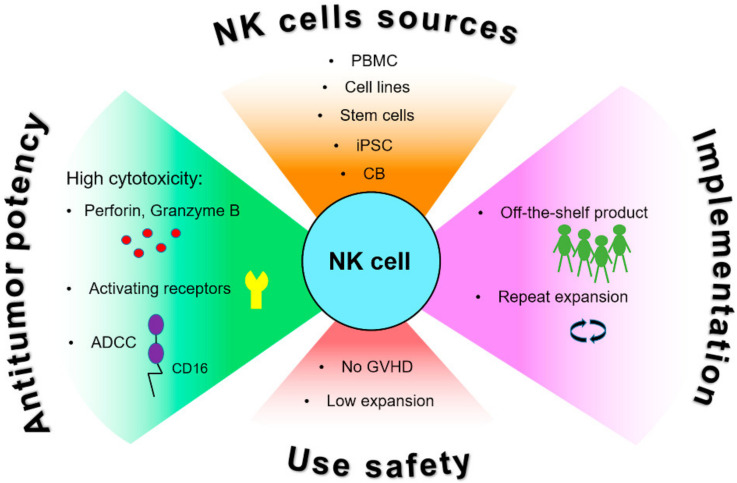
Advantages of NK cells for anti-cancer immunotherapy. NK cells have high innate cytotoxic activity caused by different mechanisms involving production of cytotoxic granules containing perforin and granzymes, multiple activating receptors and antibody-dependent cytotoxicity (ADCC) enhancing their anti-tumor potential. Different sources for obtaining and safety of use NK cells facilitate their application for research and potential clinical practice. Abbreviations: CB, cord blood; GvHD, graft versus host disease; iPSC, induced pluripotent stem cells; PBMC, peripheral blood mononuclear cells.

NK cells are lymphocytes of the innate immune system that have high cytotoxic potential against tumor cells and virus-infected cells [1,2]. Unlike T cells, NK cells do not require prior antigen sensitization or antigen presentation through HLA-I molecules to recognize their targets [3,4]. Loss of HLA-I surface expression usually caused by viral infection or cell transformation triggers NK cell activation—this fact underlies the “missing-self” principle [3]. The reactivity of NK cells depends on complex interactions between various activating and inhibiting receptors with their ligands on the surface of target cells. Activating receptors include NKG2D, DNAM-1, natural cytotoxicity receptors NKp30, NKp44, and NKp46, and a number of other receptors [5,6]. NK cells can also bind to antibodies IgG1 and IgG3 on the surface of tumor or infected cells through the CD16 receptor (also called FcγRIII) and provide antibody-dependent cell-mediated cytotoxicity (ADCC) [5]. The main inhibitory receptors are immunoglobulin-like killer cell inhibitory receptors (KIRs) and NKG2A, which belongs to the C-lectin receptor family CD94/NKG2. All of them recognize the intrinsic human leukocyte antigen class I (HLA-I) molecules. NK cells can realize cytotoxic potential against tumor cells via both the granule-mediated cytotoxicity mechanism involving perforin and granzyme B, and the receptor-mediated mechanism through cytokine TRAIL release, or the expression of surface FasL [6] (Figure 1).

## 2. NK Cells in Cancer Pathogenesis

### 2.1. The Role of NK Cells in Hematological Tumors

Hematologic tumors constitute a broad cluster of tumors that can be considered as immune cell cancers characterized by dysregulation and dysfunction of the immune system that may cause various infections or secondary malignancies. The prognostic impact of NK cells in various tumors of this category is actively studied. It has been shown that the increased number of NK cells in the bone marrow of children with acute lymphoid leukemia (ALL) upon diagnosis is positively correlated with the response to treatment and the frequency of remission [7]. The presence of active cytotoxic NK cells had a positive effect on the disease control after chemotherapy [8]. The number and functionality of NK cells was also positively associated with reduced severity in patients with chronic lymphocytic leukemia (CLL) and diffuse large B-cell lymphoma [9,10,11]. In Hodgkin’s lymphoma patients, increased infiltration of NK cells of certain phenotypes was also shown to be connected with favorable prognosis [12]. Mechanisms of the immune control of hematological tumors are closely connected with the various ways to escape from the anti-cancer immune responses realized in these tumors. Allogeneic or syngeneic NK cells are currently considered as effective agents to fight hematological tumors. The infusion of large numbers of expanded NK cells has been shown to be feasible and safe for enhancing their activity in preclinical studies and also in vivo for patients with different types of hematological tumors [13,14,15].

The studying of NK cell functional features can help to improve the knowledge about intricate NK cell effect in different malignancies. It was shown that a high level of IFN-γ secretion by NK cells is positively correlated with the therapeutic response in chronic myeloid leukemia (CML) [16], while a decrease in the secretory function of NK cells was associated with the high risk of myelodysplastic syndrome development [17]. Despite the fact that innate deficiency of NK cells is a rather rare phenomenon [18], it is most often associated with the increased risk of lymphoproliferative disease development [18,19]. Germline mutations of the perforin gene (*PRF1*) are often found in patients with childhood anaplastic large cell lymphoma and acute lymphoblastic leukemia [20,21]. Similarly, a significant proportion of adult patients with Hodgkin’s lymphoma have mutations in *PRF1* and Fas ligand genes, which are associated with the lack of NK cell activity [22]. Still, genetically determined factors are not a widespread cause of NK cell functional activity disruption, and, apparently, impaired NK cell anti-tumor response is mostly linked to phenotypic changes or the impact of extracellular factors.

#### 2.1.1. Phenotypic Features and Functional Activity of NK Cells in Hematological Tumors

The phenotypic characteristics of NK cells can influence their ability to eliminate tumor cells. As described above, the functional activity of NK cells is greatly regulated by the signal balance between activating and inhibiting receptors. Currently, natural cytotoxicity receptors (NCRs) and their ligands (NCR-Ls) are being actively studied as biomarkers for cancer patients. The main NCRs belong to the family of germ-line-encoded Ig-like transmembrane (TM) receptors, which include NKp46, NKp30, and NKp44 [5,6]. NCRs play an important role in the recognition and elimination of tumor cells in various types of leukemia [23,24,25]. The correlation between decreased NCR expression (NKp30, NKp44, NKp46) and poor prognosis and lower survival for patients with AML and CLL has been shown in different studies [26,27,28]. The tumor microenvironment (TME) plays an important role in regulating NK cell activity. For example, it has been repeatedly shown that the inhibition of the NCR and NKG2D activity against hematopoietic cancer lines can be caused by soluble factors produced in the TME including indoleamine 2,3-dioxygenase (IDO) [29] and transforming growth factor-beta (TGF-β) [30], respectively.

Despite the fact that many NCR-Ls have already been described and characterized for their functional properties, their significance for hematological tumors remains uncertain [31,32,33]. As is already known, canonical NCR ligands induce activation of NK cells; however, there are non-typical ligands called “trap ligands”, secreted in soluble form, which bind to NCRs and can inhibit cytotoxic activity of NK cells [34,35,36]. There is less data on the prognostic effect of NCR-Ls for hematological tumors than for solid tumors, probably due to less-developed mechanisms of immune response escape. Nevertheless, it has been shown that the level of soluble ligands for NKp30—BAG6/BAT3 in the plasma of patients with chronic lymphocytic leukemia is positively correlated with the stage of the disease [37]. The significance of soluble ligands has also been demonstrated for another receptor, NKG2D. The activating receptor NKG2D is a transmembrane protein from the CD94/NKG2 family of C-type lectin receptors, expressed mainly on NK cells and a part of cytotoxic T lymphocytes, such as γδT cells and CD8+ αβT cells [38]. NKG2D ligands (NKG2D-Ls) are homologues of MHC class I and are divided into two families: MIC(A,B) and RAET1/ULBP [38]. Their expression on the cell membrane is induced by stress and malignant transformation. In patients with ALL, AML, CLL, and CML, the increased level of soluble NKG2D-Ls and the decreased level of surface NKG2D-Ls were demonstrated. This was accompanied by a decrease in functional activity of NK cells and, as a consequence, worsening of the disease prognosis. This observation corresponds to the knowledge about the role of NKG2D-mediated antitumor immune response and mechanisms of tumor escape from immune response [39]. Elevated levels of soluble NKG2D-Ls, as well as reduced levels of NKG2D expression were also associated with disease progression in patients with CLL [40,41]. On the other hand, it was recently shown that Mult-1, a high affinity ligand for NKG2D, can cause NK cell activation and tumor rejection [42].

In general, the regulation of NK cell functionality via influence of activating/inhibitory receptor repertoire and soluble NK cell receptor ligands is considered as a key mechanism for tumor escape from NK cell surveillance in hematological tumors.

#### 2.1.2. Immune Checkpoint Molecules

Immune checkpoints, which are responsible for maintaining the homeostasis of an immune response, can also have an impact on the antitumor response in hematological malignancies. Various immune checkpoint molecules are presented on the surface of NK cells, such as PD-1, TIGIT, CD96, TIM-3, LAG-3, and others [43,44,45].

Increased expression of PD-1 on the surface of NK cells has been demonstrated in various hematological malignancies; this fact is important for the effectiveness of anti-PD-1/PD-L1 therapy [46,47,48,49]. Increased expression of TIM-3 was associated with a worse prognosis in patients with CLL [50]. It has also recently been demonstrated that blocking of the inhibitory molecule TIGIT can prevent NK cell depletion leading to increased antitumor response [51]. The NKG2A receptor was recently described as another potential target for immune checkpoint blockade for promoting anti-tumor immunity [52,53].

Despite the fact that most of the studies demonstrate the positive role of NK cells against hematological malignancies, some points remain unclear, for example, the effect of the quantitative/qualitative indicator of NK cells on the antitumor immune response in some hematological tumors. It has been shown that an increase in the activity of NK cells in multiple myeloma (MM) patients was positively associated with deceleration in disease progression and better patient survival [54]. However, it was previously shown in another study that a high number of NK cells is correlated with the progression of MM stage and worse survival prognosis [55]. In these patients, CD57^+^CD8^−^ cells comprised most of the analyzed NK cells. These patients were characterized with a better suppression of tumor progression [55]. In another study, a high NK cell number was associated with lower survival in cutaneous T-cell lymphoma [56]. Such ambiguous data on the role of NK cells in the pathogenesis of MM and T-cell lymphoma can result from the different distribution of NK cell subpopulations between different hematological malignancies as well as from the tumor microenvironment influence that affects NK cell functional activity [54,56,57]. In addition, as long as the foci of myeloma and cutaneous T-cell lymphoma are localized in bone marrow and skin, tumor development takes place in situ and malignant processes are similar to those of solid tumors. Thus, tumor microenvironment influence is of great significance in inhibiting the antitumor response.

Mechanisms disrupting NK cell antitumor response and reducing the functional activity of NK cells have been described in different studies [58]. These include an overexpression of immune checkpoint ligands for PD-1, TIM-3, TIGIT, ligands for other inhibitory receptors impairing the immune response, and suppression of the NCRs (NKp30, NKp44, and NKp46) [26,27,28] both by reducing their expression and binding with soluble NCR-Ls. However, the effects of all these mechanisms in different types of hematological malignancies still have to be studied.

Despite the existing gaps, the data obtained in biological experiments significantly correlate to and complete wide range of clinical data. Restoring and/or enhancing the functional activity of NK cells is one of the main strategies for therapeutic approaches to the treatment of hematological tumors.

### 2.2. The Role of NK Cells in Solid Tumors

Despite the rather extensive knowledge about the functional role of NK cells in the antitumor immune response in hematological tumors, relevant knowledge in the context of solid tumors is still limited. Studies using animal models have shown that depletion of NK cell populations prior to tumor transplantation leads to more progressive development of a number of solid tumors with metastatic progression [59,60]. The use of mutant mice with alterations in the development or functional activity of NK cells made it possible to better understand the role of these cells in antitumor immunity. For instance, the model of NKp46-/- knockout mice allowed us to find out that NK cells are directly involved in the destruction of cancer cells in melanoma and metastatic lung carcinoma [61]. A similar study showed that a lack of important adhesion glycoprotein of NK cells known as DNAM-1 impaired their functionality. It was shown that DNAM-1-/- knockout mice had decreased NK cell cytotoxic function compared to WT against a fibrosarcoma cell line [62]. 

However, there is still no clear understanding of the degree of NK cell immune response impact in different types of solid tumors. Early studies demonstrated that low NK cell infiltration predicts increased colorectal cancer severity and recurrence [63,64], but the results confirmed this fact still remains inconsistent [65,66,67]. Recently, it was shown that colorectal tumors had a low infiltration of NK cells, despite the high concentration of chemokines promoting NK cell infiltration [68]. Robust expansion and cytotoxicity of CD56^dim^CD57^dim^KIR^+^ NK cells were demonstrated against tumor cells in patients with melanoma [69]. At the same time, NK cells from metastatic lymph nodes (MLN-NK) have decreased cytotoxicity [69]. In another similar study, it was shown that inactive MLN-NK were presented by a subset of CD56^bright^CD16^+^ cells and NKp30 and NKG2D expression levels were negatively correlated with the number of tumor cells in the infiltrated lymph nodes in metastatic melanoma [70], indicating the possible reason of decreased MLN-NK cells cytotoxic activity.

Yet, active participation of NK cells in anti-tumor response demonstrated different solid tumors. In patients with breast cancer, increased intratumoral NK cell infiltration was associated with better prognosis and lower mortality compared to patients with low NK cell infiltration [71]. An increased level of tumor-infiltrating NK cells is associated with better prognosis for many solid tumors, including gastrointestinal stromal tumor [72,73], neuroblastoma [74], hepatocellular carcinoma [75], glioblastoma [76], head and neck cancer (HNC) [77] and prostate cancer [78].

#### 2.2.1. Phenotypic Features and Functional Activity of NK Cells in Solid Tumors

The impact of infiltrating NK cells’ phenotype on their functional activity appears to be high. The decreased expression level of the activating receptors NKp46, NKp30, and NKG2D in peripheral blood NK cells in patients with cervical cancer is correlated with tumor progression [79]. Analogically to hematological tumors, the production of “trap-ligands” for NCR can inhibit NK cell cytotoxic functional activity. For example, the presence of the soluble form of B7-H6 (sB7-H6), the NKp30 ligand, has been detected in the peritoneal fluid of patients with ovarian cancer, metastatic gastrointestinal stromal tumor, neuroblastoma and hepatocellular carcinoma [35,74,80,81]. Notably, the role of soluble ligands in controlling NK cell functionality is not always negative. It was shown recently that platelet-derived growth factor (PDGF)-D, a soluble ligand for NKp44, can promote activation of NK cells and control dissemination and growth of different types of solid tumor cell lines [82]. In turn, nidogen-1, another soluble NKp44 ligand, which has been found in high levels in the sera of patients with ovarian and lung cancer [83,84], has been shown to inhibit PDGF-D-induced activation of NK cells [36]. The PDGF-D mediated pathway of NK cell activation has recently been associated with anti-tumor immunity [85]. Apparently, similar to surface ligand interactions, soluble ligand-derived signals contribute to the activating and inhibitory signaling balance and influence NK cell functionality.

In a clinical study of patients with kidney cancer, the phenotypic features of infiltrating NK cells were determined. Two groups of patients were identified whose NK cells differed in the level of CD16 expression. In tumors with high NK cell content (>20% of the lymphocyte population), most of the NK cells were CD16^bright^, whereas in the tumors with low NK cell count (<20%) most of the NK cells were CD16dim. In vitro experiments showed that NK cells from patients with high NK cell infiltration demonstrated cytotoxic response against K562 cells, in contrast to the group with a low NK cell count [86]. In another work, high density of NK cells in the intratumoral region in patients with hepatocellular carcinoma positively correlated with overall survival and disease-free survival; however, NK cells obtained from biopsies of progressive hepatocellular carcinoma showed decreased production of antitumor cytokines [87]. Staining for NKp46 revealed that renal cell carcinomas (RCC) and gastrointestinal stromal tumors (GIST) have strong NK cell infiltration. However, NK cells infiltrating RCC demonstrated no cytotoxicity and upregulated NKG2A inhibitory receptor expression [88]. NK cells infiltrating GIST tumors expressed immunosuppressive NKp30c isoform, which had a prognostic role for these patients [89]. Poor infiltration of HCC by NK cells has also been reported, and few NK cells present in the tumor were functionally anergic, as indicated by diminished NKp30 and DNAM-1 expression [85]. Some of the NK cells were localized in the stroma and had no proper contact with tumor cells [90].

On the other hand, the level of NK cell infiltration did not affect clinical outcome in non-small cell lung cancer (NSCLC) and lung adenocarcinoma [91,92]. Interestingly, clinical outcomes of patients with NSCLC depended more on the phenotype and functional activity of NK cells rather than on their density in the tumor foci [93]. Still, NSCLC-infiltrating NK cells were found to have severely impaired cytotoxic potential, despite the fact that they expressed several activation markers including NKp44, CD69 and HLA-DR. The expression of activating receptors such as NKp30, NKp80 and CD16 was significantly reduced in these cells [92,93,94]. Lung biopsies obtained from NSCLC patients revealed higher levels of CD56^bright^CD16^−^CD57^−^ NK cells inside the tumor than in the surrounding normal lung tissue. These NK cells also expressed inhibitory KIRs normally expressed on the CD56^dim^CD16^+^ subpopulation. The expression level of PD-1 and CD96 were increased too in the tumor-infiltrating NK cells compared to NK cells in the surrounding tissues and circulating cells [92,94].

Phenotypical changes in NK cells can affect their effective migration to tumor foci, limiting antitumor activity against solid tumors. Transcriptomic analysis of intratumoral NK cells from NSCLC biopsies has revealed significantly reduced expression of sphingosine-1-phosphate receptor 1 (S1PR1) and CX3C chemokine receptor 1 (CX3CR1)—important receptors participating in the migration of NK cells into the tumor [95]. The ability of NK cells to penetrate into primary tumors and migrate to metastatic sites largely depends on the enzyme heparanase involved in the degradation of the polysaccharide heparan sulfate—key components of the intercellular substance in the connective tissue. Mice knocked out for heparanase in NK cells were more susceptible to the development of lymphoma, metastatic melanoma, prostate carcinoma, or breast cancer after intravenous administration of the carcinogen methylcholantrene [96].

**Figure 2:**
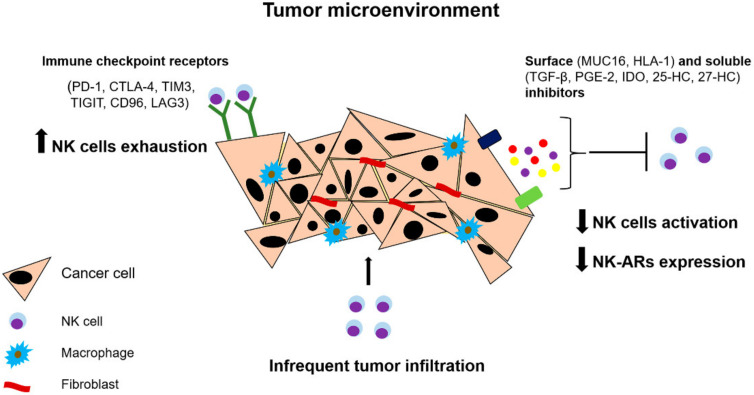
Influence of microenvironment of solid tumors on NK cell functionality. The tumor microenvironment promotes reducing NK cell functionality via production of different soluble and surface inhibitors negatively affecting activation, maturation, proliferation and effector functions of NK cells. Infrequent tumor infiltration decreases the chance of NK cells to migrate in tumor foci. Abbreviations: PD-1, programmed cell death 1; CTLA-4, cytotoxic T-lymphocyte-associated protein-4; IDO, indoleamine 2,3-dioxygenase; HLA-1, Human leukocyte antigen 1; LAG3, Lymphocyte-activation gene 3; MUC16, Mucin 16; NK-ARs, activating NK cell receptors; TGF- β, transforming growth factor-β; PGE-2, prostaglandin E2; TIGIT, T cell immunoreceptor with Ig and ITIM domains; TIM3, T-cell immunoglobulin domain and mucin domain 3; 25-HC, 25-hydroxycholesterol; 27-HC, 27-hydroxycholesterol.

Generally, NK cells can penetrate solid tumors with varying success. However, in most cases, antitumor properties of NK cells are weakened when they enter the TME. TME includes cancer cells, fibroblasts, endothelial cells and various immune cells, including cells which provide conditions for tumor escape from the immune response. Phenotypical changes in NK cells caused by the influence of TME often underlie the reasons of NK cell anergy and dysfunction (Figure 2).

#### 2.2.2. The Role of Immunosuppressive Molecules in NK Cell Functioning

Both surface-expressed and soluble modulators produced in the TME can negatively regulate the maturation, proliferation, and effector function of NK cells. These immunosuppressive factors can act either directly on NK cells or indirectly by activating other immune cells, such as antigen-presenting cells (APC), regulatory T cells, and myeloid suppressor cells, by stimulating production of additional immunosuppressive molecules [97]. For example, neuroblastoma cells can modulate chemokine receptor repertoire of NK cells by producing TGF-β [97,98]. TGF-β is able to reduce the recruitment of CD56^dim^ NK cell subpopulation and favors the migration of less cytotoxic CD56^bright^ NK cells. It also promotes the modulation of chemokine receptor repertoire by decreasing the expression of the chemokines that attract CD56^dim^ NK cells (CXCL2, CX3CL1, CXCL1, and CXCL8), and increasing the expression of chemokines stimulating the migration of CD56^bright^ NK cells (CXCL9, CXCL10, CCL5) [98]. In colorectal and lung cancer, decreased expression of NKG2D was associated with increased level of TGF-β in the blood serum of patients [99]. Currently, suppression of TGF-β signaling is actively studied and applied in preclinical studies, including combination therapy using checkpoint inhibitors such as anti-PD L1 drugs [100,101].

An important factor causing functional disorders in NK cells is the disruption of their cellular metabolism [102]. A recent study has shown that, in lung tumors, the expression of the gluconeogenesis enzyme fructose 1,6-bisphosphatase in tumor-infiltrating NK cells is enhanced via the mechanism involving TGF-β. This leads to dysfunction of NK cells through inhibiting the process of glycolysis, which is highly important for the activation of NK cells [103]. Another physiologically active compound potentially capable of inhibiting NK cell metabolism includes two components of enzymatic cholesterol oxidation: 25-hydroxycholesterol and 27-hydroxycholesterol. They both can inhibit the activation of SREBP transcription factors—key regulators of the lipid metabolism [104,105,106]. Additional data supporting the role of impaired cellular metabolism in the development of NK cell dysfunction has been obtained from studies using mice models of obesity. It has been found that NK cells from obese mice are less sensitive to tumor cells due to inhibition of their cytotoxic activity and decreased production of IFN-γ, granzyme B, and perforin [107]. Increased production of prostaglandin E2 (PGE2) in the tumor microenvironment also suppresses the effector function of NK cells. PGE2 produced by the microenvironment of thyroid cancer cells suppresses NK cell cytotoxicity. Preclinical studies have demonstrated that the inhibition of PGE2 in a mouse model of metastatic breast cancer and in a model with a human gastric cancer cell line restored cytotoxic function of NK cells [108,109].

Another mechanism by which tumor cells avoid NK cell attack is by preventing cell contact. Ovarian tumor cells impair the formation of immunological synapses with NK cells by expressing the glycoprotein mucin 16 (MUC16), which has anti-adhesive properties [110]. Tumor cells can also interfere with NK cell activation by inhibiting the expression of ligands for activating receptors. For example, melanoma cells become resistant to NK cell attacks by increasing their surface expression of both classical and non-classical class I HLA molecules [111]. NK cell activity can also be suppressed by the interaction of NKp44 receptor with proliferating cell nuclear antigen (PCNA), which is overexpressed on the surface of many types of tumor cells [112,113]. In addition, increased expression of the ligands for checkpoint inhibitors, such as PD1, CTLA4, TIM3, TIGIT, CD96, KLRG-1, and LAG3, on the tumor cells attenuates antitumor activity of NK cells [114]. This fact is reflected in the development of a whole new section in the modern approaches to immunotherapy—checkpoint inhibitors [115,116]. The prospective studies include novel targets for therapeutic approach. It was shown that non-typical immune checkpoint regulator interleukin-1 receptor 8 (IL-1R8) can regulate anti-tumor response via NK-cell mediated resistance to hepatic carcinogenesis and hematogenous liver and lung metastasis [117].

As is shown above, the main limitations of NK cell functional activity in the context of the immune response to solid tumors are associated with two factors: (1) the problem of the impaired migration of functionally active NK cells into the tumor site; (2) direct immunosuppression by tumor microenvironment in situ: it often plays the most important role in the development of resistance. Based on the current knowledge about the effect of TME on NK cell response, it can be assumed that the second factor is the key one and is prevalent among the solid tumors compared with hematological tumors, due to more complex organization of the solid tumors in situ. Obviously, further studies and clinical research, including NK-cell-based therapies, will be aimed at overcoming these limitations.

## 3. Current Approaches to NK-Cell-Based Immunotherapy

### 3.1. Hematological Tumors

Current immunotherapy approaches for cancer treatment are aimed at restoring or enhancing the ability of NK cells to attack cancer cells, including hematological malignancies. Major therapeutic strategies include: (1) hematopoietic stem cell transplantation (HSCT)—a therapeutic strategy that exploits the alloreactivity of NK cells. This strategy has an improved analogue—adoptive transfer of isolated NK cells, where NK cells can be pre-multiplied, activated, and/or genetically modified; (2) the use of monoclonal antibodies (mAbs) for: (a) enhancing the signal from the activating NK cell receptors; (b) activation of ADCC; (c) inhibition of the surface receptors and ligands of immune checkpoints; (3) the increase in NK cell activity of using cytokines and other immunostimulating drugs (Figure 1).

#### 3.1.1. Hematopoietic Stem Cell Transplantation in Treatment of Hematological Malignancies

Currently, HSCT and the adoptive transfer of NK cells are actively developing approaches in immunotherapy [118,119]. Allogeneic bone marrow stem cell transplantation is sometimes a life-saving treatment for patients suffering from highly progressive malignant hematological diseases. Due to the multiple differences between HLA-I and HLA-II between the donor and the recipient, bidirectional alloreactivity caused by incompatible HLA molecules can lead to serious clinical complications including graft rejection and incidence of acute and chronic graft versus host disease (GvHD). Allografts based on T cells are still the most-used transplantation products and are usually responsible for the occurrence of severe GvHD [120]. Thus, the search for various strategies aimed at avoiding negative consequences has led to studies considering the possibility of using NK cells for transplantation [121,122]. NK cells look even more attractive for therapeutic applications due to the fact that they are the first cells from the lymphocyte pool which recover after HSCT [123].

The advantage of using NK cells over T cells is the reduced risk of a graft versus host reaction as NK cells mostly do not attack cells expressing HLA class I molecules. Still, there might be a selection step before using allogeneic NK cells—KIRs-HLA-I typing aims to find mismatches in KIRs expressed on the donor NK cells and HLA-I expressed on the host cells. Low expression of HLA-I in general or normal expression of the donor’s HLA alleles which are not recognized by KIRs of the recipient is the factor of positive selection for alloreactive NK cells. This process is reflected in vivo in the so-called “licensing” of NK cells via recognition and “memorization” of the interactions between HLA-I and KIRs [124]. However, only 25% of patients who need an allograft find an HLA-identical donor. This fact complicates the implementation of this therapeutic approach to treatment. Nevertheless, there are many studies confirming the effectiveness of NK cell transplantation in the treatment of hematological cancers. A favorable effect of the use of haploidentical NK cells donor has been observed in children with ALL, demonstrating a 70% survival rate versus 35% in patients who did not receive alloreactive NK cells [115]. The studies using haploidentical bone marrow transplants with depleted T cells for AML treatment have shown that patients who lacked the KIR ligands (HLA-I alleles) presented in haploidentical donors had a reduced risk of leukemia recurrence (75% versus 0% after 5 years) and did not acquire GvHD while demonstrating increased overall survival [125]. It has also been shown that using HSCT with depleted CD3^+^CD19^+^ cells led to better engraftment and restoration of stable immune response [126,127]. NK cell transplantation has been used in both children and adults with ALL and AML, but the effectiveness of this therapy for AML was often limited even after KIR-HLA typing [121,128,129,130]. Possibly, the effectiveness of NK cells depends on their source and/or on the purity of T cell depletion for the prevention of GvHD.

#### 3.1.2. Combination Therapy with NK Cell Transplantation

Combining different therapeutic approaches with NK cell transplantation is a popular research area nowadays. Many studies are aimed at the development of the ways to increase activity and persistence of the injected NK cells. The first phase I/II clinical trials on the use of recombinant human IL-15 (rhIL-15) administered subcutaneously or intravenously together with the injection of haploidentical NK cells after lymphodepletion in patients with relapsed or refractory AML have demonstrated positive therapeutic response: rhIL-15 induced the growth of NK cells in vivo and increased the rate of remission. The effectiveness of rhIL-15 was significantly higher compared to the use of IL-2 [131]. In another phase I clinical trial NK cells isolated from the HLA-haploidentical donors were pre-activated with a lysate of leukemia cell line CTV-1 CNDO-109, which resulted in increased cytotoxicity and activation of NK cells in AML patients [132].

There are also a number of studies estimating the combination of HSCT and NK cell transplantation. Although the efficacy remains controversial, NK cell transplantation before or after HSCT was shown to be applicable without serious side effects [133,134,135]. A phase I/II clinical trial in which AML patients received haploidentical HSCT combined with early adoptive NK cell transfer demonstrated 37% of two-year overall survival compared to 14% for patients who underwent haploindentical HSCT, suggesting that transferred NK cells may contribute to long-term remission in patients with refractory AML [136]. Although NK cell adoptive transfer appears to be a rather safe procedure, clinical responses are still limited, and this fact requires further study.

#### 3.1.3. Genetic Engineering of NK Cells

Genetic engineering offers another strategy to increase the antitumor potential of adoptively transferred NK cells. Chimeric antigen receptors (CARs), initially created for T cells, demonstrated impressive clinical results in ALL and NHL and were a significant breakthrough in the therapy of hematological tumors [137]. Adoptive cell therapy based on the use of CAR-engineered T cells has achieved impressive results for B-cell lymphoma and leukemia [138,139]. Two types of CARs based on antigen receptors towards CD19 have been approved for CAR-T therapy, first by the FDA and then by the European Medical Agency, for the treatment of recurrent B-cell ALL and refractory/recurrent large cell non-Hodgkin’s lymphoma (NHL). However, CAR-T cells have a number of limitations, namely: (1) high cost of production; (2) significant time lag between the identification of the patient’s receptor repertoire and the therapy itself; (3) lack of possibility for multiple repeated administration of the drug to the patients who have low persistence of CAR-T cells or disease relapse; (4) a high level of toxicity is observed in most of the patients due to the production of IFN-γ and, as a consequence, the induction of cytotoxic syndrome and/or neurotoxicity. In theory, NK cells could be an alternative source for CAR modification. CAR-NK cells are considered a safer product than CAR-T cells because: (1) NK cells have a lower risk of developing a graft versus host reaction; (2) they are short-lived cells with low expansion, and despite the fact they also produce cytokines such as IFN γ and GM-CSF, the risk of developing “cytokine syndrome” is lower than for T cells; (3) NK cells are supposed to show high innate cytotoxic activity due to (a) multiple activating receptors enhancing the activity of CAR; (b) antibody-dependent cytotoxicity making it possible to combine this therapy with mAbs; (4) they give a possibility of creating a “bank” of allogeneic NK cells; (5) they are cheaper in production and can be obtained from various sources including NK cell lines, umbilical cord blood (UCB), and induced pluripotent stem cells (iPSC). Such a large number of sources results in a great number of preclinical and clinical studies using different combinations of CAR-NK cells with other therapeutic approaches. Currently, CAR-NK cells targeting several antigens including CD19, CD20, CD33, CD138, CD3, CD5, CD7, CD123 and others, obtained from both primary NK cells and NK-92 cell line, are being investigated in preclinical and clinical trials [140,141,142,143,144,145]. A recent phase I clinical trial using CD33-targeted CAR-NK-92 cells for the patients with recurrent refractory AML has shown no serious side effects, indicating that CAR-NK cells may be a safer alternative to CAR-T cells [146]. Recently, an unmodified NK-92 cell line was approved by the US FDA as a source of NK cells for clinical trials. This line is irradiated before the adoptive transfer to prevent the development of NK cell-malignancies in patients, and it has proven its safety in phase I/II clinical trials [147,148,149]. However, the clinical use of NK-92 is currently limited. So far, data are available from only one clinical study using NK-92 cells with CAR-CD33 in three patients with recurrent and refractory AML. This study has shown that doses up to 5 × 109 irradiated cells per patient did not cause significant side effects along with a marginal and transient patient response [146]. To date, an attempt to optimize CAR-NK-92 approaches has been made in several clinical trials that include CAR-modified NK-92 to target BCMA in MM(NCT03940833) and CD19 in leukemia and lymphoma (NCT02892695).

Primary NK cells derived from umbilical cord blood have also been studied as a possible platform to create CAR-NK for several reasons: (1) low risk of virus transmission from the donor to the recipient; (2) high concentration and, as a consequence, high availability of NK cells (“finish” product is quickly obtained); (3) less stringent HLA matching requirements and lower risk of GvHD [150]. Several models have been developed in vitro for optimization and large-scale expansion of UCB-NK cells using feeder cells expressing co-stimulatory molecules, such as membrane bound IL-21 or IL-15 [151,152]. To improve the expansion and stability of NK cells, an optimized CD19-based CAR construct was developed for NK cells with additional expression of IL-15. In a preclinical trial, strong persistence and increased antitumor activity of the CD19-CAR-IL15-modified NK cells have been demonstrated [153]. A phase 1/2 clinical trial using this model is currently underway (NCT03056339).

#### 3.1.4. NK Cells and Cytokine-Based Therapy

Another key therapeutic strategy aiming at increasing the antitumor activity of NK cells is cytokine stimulation. Cytokines have been considered as promising agents for cancer treatment for a long time. However, their pleiotropism has complicated their clinical use. This fact probably reflects used strategies of development in the treatment area. For example, stimulation of NK cells with a combination of IL-15, IL-12, and IL-18 before the adoptive transfer has demonstrated promising results: it was shown that ex vivo cytokine-treated NK cells persisted longer and reminded cytokine-induced memory-like (CIML) NK cells of their adaptive-like properties characterized by increased IFN-γ production and cytotoxicity against leukemia and lymphoma cells [154,155]. In a preclinical study, human CIML NK cells also demonstrated high cytotoxicity against myeloid leukemia cells in vitro and in vivo, suggesting that they could be potentially applied in therapy [156]. A first-in-human phase I clinical trial demonstrated CIML-NK cell expansion and stable responses against AML blasts [157].

Several cytokines promote survival, proliferation, differentiation, and activation of NK cells. This list includes interleukins (IL)-2, -15, -12, -18, -21 enhance anti-tumor function of NK cells and increase proliferation in vitro and in vivo [158,159].

IL-2 is still one of the most popular cytokines used to enhance the cytotoxicity of NK cells [159]. However, IL-2 therapy can lead to severe adverse effects such as vascular leakage or organ injury caused by binding IL-2 high affinity receptor IL-2Rαβγ expressed on the vascular endothelium [160]. Moreover, Treg cells also express IL-2Rαβγ receptors and can be activated by IL-2, inhibiting NK cell proliferation and cytotoxicity [161]. Both these facts described above resulted in the development of a mutant form of IL-2 called “super-2”. “Super-2” was made with increased affinity for the IL-2/15Rβ subunit present on NK cells, but low affinity for the IL-2Rα subunit IL-2Rαβγ [162]. Along with “super-2”, another mutant forms of IL-2 with high affinity for IL-2Rβγ present on NK cells and reduced affinity for IL-2Rα expressed on Treg cells were shown to be capable of promoting NK cell activation [162,163,164].

IL-15 uses common receptor subunits with IL-2, but unlike IL-2, it does not induce Treg-mediated immune suppression [165]. Mainly, the clinical application of IL-15 is restricted due to a short lifetime. The use of a formed construct of IL-15 with IL-15Rα (named IL-15 superagonist) led to a longer half-life and higher biological activity than native IL-15 [166]. In the first clinical trial, recombinant human (rh) IL-15 was used in patients with metastatic malignancies. Despite the fact that NK cell activation and proliferation was observed, objectively no responses to therapy were detected [167]. Nowadays, several clinical trials (NCT03388632, nivolumab and ipilimumab; NCT02689453, alemtuzumab; NCT03759184, obinutuzumab; NCT03905135, avelumab) are evaluating therapeutic use of rhIL-15 in combination with antibodies.

ALT-803 is a superagonist of IL-15 containing IL-15Rα fused to IgG1Fc which is bounded to IL-15 mutein (N72D). The improvement of disease prognosis has been observed in a first phase I clinical trial study after ALT-803 IL-15 (superagonist) administration in patients with hematologic malignancies who had relapse after allogeneic HSCT. It has been shown in a preclinical study that ALT-803 can both stimulate innate and adaptive immune responses by enhancing NK cell functions with no severe side effects and GvHD [168]. The effectiveness of the use of ALT-803 is being evaluated in combination with antibodies (NCT02384954) and adoptive NK cell transfer (NCT01898793, NCT02782546).

Another method to improve HSTC results consisted of the expansion of NK cells ex vivo by genetically modified K562 feeder cells with membrane-bound IL-21 (K562-mbIL21) [169]. Recently, this method has been used in a phase I clinical trial with haplo-HSCT. It appeared to be safe and effective for treating leukemia, with significantly increased NK cell number and functional activity, low toxicity, and low post-transplant relapse rate [170].

#### 3.1.5. NK Cell-Directed Monoclonal Antibodies

In addition to NK cell transplantation, the use of mAbs is also a promising and actively developing area of NK cell therapy. The use of tumor-specific chimeric mAbs that promote ADCC by ligating CD16 receptor on NK cell has become the therapeutic standard for many hematological malignancies. CD16a/FcγRIIIA is expressed on most NK cells, and it is the only activating receptor capable of triggering cytotoxic activity of NK cells even in the presence of inhibitory signals. One of the first drugs from this group used in clinic is rituximab—a monoclonal antibody with specificity for the CD20 antigen, and it is still the first-line drug for the treatment of B-cell chronic lymphocytic leukemia [171]. In addition to the “classic” rituximab, other mAbs have confirmed their effectiveness and have been launched into therapeutic practice. These include obinutulumab, which has an affinity for CD20 as well as rituximab, daratumumab, which binds the CD38 antigen, and elotuzumab, which binds SLAM7.

Bispecific antibodies (BiKEs) are probably the most promising of the antibodies. BiKEs link two antigen-binding domains of two antibodies into a unique molecule which targets two epitopes simultaneously. The first “bispecific T cell engagers” (BITEs) were developed to redirect T cell-mediated cytotoxic activity against transformed B cells [172]. A new generation of BiKEs and trispecific antibodies (TRiKEs), which stimulate NK cells, have been developed recently. They can activate NK cells against one or more tumor antigens via CD16-mediated ADCC. For now, few clinical reports are available, as BiKEss and TRiKEs directing NK cells have only recently begun to be applied in clinical applications. In the existing studies, they demonstrate significant efficacy and stable safety profile in the initial clinical and late preclinical phases [173]. They have been shown to increase NK cell-mediated cytotoxicity and cytokine production in leukemia and lymphomas [174]. It has also been demonstrated that bispecific antibody targeting CS1-NKG2D prolonged survival in a mouse xenograft model against MM cell line [175].

A promising therapeutic approach to the treatment of patients with leukemia is the use of monoclonal antibodies capable of disrupting interactions between the immune checkpoints and other inhibitory receptors expressed on NK cells and their ligands expressed on the tumor cells, and thus restore the effective NK cell mediated antitumor response. Receptors inhibiting the activity of NK cells belong to one of the four categories: (1) killer immunoglobulin-like receptors (KIRs); (2) lectin type c-receptors (NKG2A/CD94,); (3) leukocyte immunoglobulin-like receptors (LILRs); and (4) immune checkpoint receptors (PD-1, TIM-3, LAG-3 and TIGIT). Immune checkpoint inhibitors have found wide applications in clinical practice, in particular anti-PD-1, anti-CTLA-4, anti-TIM-3 antibodies, and so on. At the moment, PD-1 inhibitors (nivolumab, pembrolizumab) are actively used in clinical practice for the treatment of Hodgkin’s lymphomas and diffuse B-cell lymphoma [176]. Many studies are being carried out involving other inhibitory molecules. It has been shown that the mAb lyrilumab, directed against common epitope for KIR2Ds, efficiently blocked KIR/KIR-L interaction and increased NK cell-mediated destruction of blast lymphoid cells both in vitro and in vivo [177]. In clinical trial, the use of anti-KIR mAb IPH2101 at a minimum dosage of 1 mg/kg in AML patients with complete remission have shown KIR-mediated binding to more than 90% of NK cells (NCT01256073). Treatment with anti-KIR antibody has resulted in increased serum concentrations of TNF-α and MIP-1β, as well as increased expression of CD69, known as an early NK cell activation marker [178]. Another mAb used in immunotherapy to restore NK cell function is represented by anti-NKG2A monalizumab (IPH2201) blocking the NKG2A/HLA-E interaction. It has been previously shown that blockade of NKG2A in patients with CLL can lead to the restoration of the functional activity of NK cells [179]. The possibility of using IPH2201is being tested in clinical trials for various types of hematological tumors (NCT02921685).

### 3.2. Solid Tumors

Despite the widespread use of NK cells in the therapy against hematological malignancies, similar advances in solid tumors are still limited. The functional response of NK cells to solid tumors is less efficient in the context of therapeutic use than to hematological tumors, and current research in this area aims to solve this problem. As we mentioned above, there are two major reasons for the limited use of NK cells in solid tumor therapy: (1) the difficulty of maintaining long-term persistence of activated NK cells in the TME due to its inhibitory effect; (2) the difficulty of achieving effective migration of NK cells inside the tumor body [180,181].

In solid tumor TME, NK cells need to overcome a number of immunosuppressive factors. A recent phase I clinical trial has demonstrated treatment resistance in 8 out of 17 patients with lymphoma and solid tumors after three allogeneic NK cell transplants. The level of NKG2D-positive T cells was increased significantly along with the reduced frequency of Treg, myeloid suppressor cells (MDSC) and TGFβ production, suggesting that the injected NK cells can enhance the activation of T cells while functional activity of NK cells is impaired [182]. TME factors inhibiting activity of NK cells usually include IL-10, TGFβ, IL-6, PGE2, indolamine-2,3-dioxygenase (IDO), adenosine, and hypoxia stress [183,184]. Using a mouse model, it has been shown that melanoma tumor cells produce PGE2, converting monocytes into suppressor cells of myeloid origin (MMP), which strongly inhibit activity of NK cells via stimulation of TGF-β production. Suppression of the inflammation modulator cyclooxygenase-2 (COX-2) led to a decrease in the accumulation of MMP in the peripheral blood, which caused simultaneous improvement in the clearance of tumor cells in vivo [185].

The efficiency of NK cell migration into tumors is an important parameter of antitumor response, which is controlled by chemokines and adhesion molecules. The repertoire of chemokine receptors is changed during the ex vivo expansion and activation of NK cells. For example, NK cells increase the expression of CXCR3 upon activation. This facilitates their migration along the gradients of IFNγ-induced chemokines CXCL9, CXCL10, and CXCL11. It has been shown in vivo that CXCR3-deficient NK cells cannot migrate to CXCL10-positive B16 melanoma tumor cells [186]. In a similar study using an in vivo xenograft model, it was demonstrated that activated CXCR3-positive human NK cells selectively migrate towards CXCL10-positive human melanoma tumor cells [180]. However, CXCL10 is produced only by the inflamed foci of the tumors. Solid tumors often produce high levels of IL-8, and oncogene-dependent chemokines recruit cells expressing CXCR1 and CXCR2 receptors [187]. Although peripheral blood NK cells express CXCR2 receptor, it is rapidly lost when NK cells are activated ex vivo. In a recent study, it has been demonstrated that gene modification of the activated human NK cells to overexpress CXCR2 receptor facilitated the migration of NK cells to tumor cells producing IL-8 [187]. In the same study, a molecular structure based on the antibody-bound NK cell homing protein (NRP-body) has been developed. NRP body administration increased NK cell tumor infiltration along the CXCL16 chemokine gradient in a mouse model of primary metastatic pancreatic duct adenocarcinoma. The infusion of the NRP-body improved infiltration of adoptively transferred NK cells into the tumor tissues and decreased the tumor load, thus increasing overall survival compared to the control group. Moreover, genetically modified NK cells with increased CXCR2 expression more efficiently migrated to the tumor foci of kidney cancer [187].

The influence of TME and efficiency of NK cell migration to tumor might be considered as the main limiting factors for full NK cell functionality in context of solid tumors. Most novel immunotherapeutic strategies are aimed to solve the problems of NK cell short-time persistence and activity, NK cell target delivery, and influence the factors inhibiting NK cell functions.

#### 3.2.1. Adoptive NK Cell Transfer for Treatment of Solid Tumors

Recently, much interest in the treatment of solid tumors has focused on the possibility of using the adoptive transfer of ex vivo-activated NK cells. At the moment, the efficiency of NK cell transplantation for solid tumors is limited [188,189] due to the limitations described above. One of the main strategies aiming to improve the adoptive transfer of NK cells is the use of gene modifications based on CAR technology. Currently, several CAR-NK constructs recognizing EGFR in breast cancer with metastases to the brain [190], ErbB2/HER2 in breast cancer [191,192], EGFRvIII in glioblastoma [193], GD2 in neuroblastoma [194], EpCAM in breast carcinoma [195], NKG2D in ovarian cancer [196], and MUC1 and ROBO1 in various refractory solid tumors have been developed and are already used for both preclinical studies and phase I/II clinical trials (Table 1).

An attempt to genetically modify NK cells based on their existing costimulatory structures opens up great opportunities for new research. A chimeric molecule based on the extracellular region of the inhibitory PD-1 receptor and transmembrane domain of the activating NKG2D receptor has been designed to help NK cells escape from the immune suppression mediated by PD-1 ligands expressed on solid tumor cells. The construct included the chimeric PD1-NKG2D receptor containing the NKG2D hinge region and the 4-1BB costimulatory domain. Stable surface expression of this construct was obtained on NK-92 cells, which further showed an increased level of cytotoxicity against various solid tumor cell lines in vitro [197]. In other, similar studies, DAP12 signaling molecule was used in CAR construction. In two independent studies, two variants of CAR have been created: (1) the intracellular signaling molecule DAP12 was cross-linked with the anti-PSCA single-chain Ab fragment scFv(AM1), resulting in the increased cytotoxicity of the YT NK cell line against PSCA-positive tumor cells compared to CAR-NK-containing CD3ζ signaling domain [198]; (2) DAP12 was cross-linked to the extracellular domain of NKG2D [199]. The last study is of particular interest because it has been tested in a pilot clinical trial with three chemotherapy-resistant patients with metastatic colorectal cancer to assess the safety and feasibility of adoptive cell therapy with primary NK cells. The patients have received multiple infusions of autologous engineered CAR-NK cells (first patient) or allogeneic CAR-NK cells from HLA-haploidentical family donors (second and third patients). Importantly, it was reported about only one manifestation of grade 1 cytokine release syndrome (CRS), associated with fever and fatigue, by the first patient, whereas in two patients treated with haploidentical CAR-NK cells, GvHD and CRS were not observed [199].

Activated and expanded NK cells can be obtained from different sources. Recently, it has been shown that NK-92 cells can be engineered to express T cell receptors (TCR) directed against melanoma antigens [200,201]. NK-92 cell line was used for obtaining high-affinity CAR-NK (haNK) cells expressing CD16A and IL-2. Obtained chimeric haNK cells demonstrated a high level of granzyme and lysis of a spectrum of human solid tumor cells [202] and were approved for use in patients with different solid tumors (Table 1). There has been increasing interest in NK cells derived from induced pluripotent stem cells (iPSC-NK) or human embryonic stem cells (hESC-NK). It was shown that the advantage of iPSC-NK cells is the possibility of providing a homogeneous NK cell population that can help to standardize cell growth for clinical use [203,204]. Using a murine ovarian cancer xenograft model, Li et al. have shown that NK-CAR-iPSC-NK cells directed against the mesothelin antigen inhibited tumor growth and increased survival significantly more compared to peripheral blood NK cells, iPSC-NK cells, or T-CAR-iPSC-NK cells (CAR containing CD28 transmembrane and co-stimulatory domains) [196]. Moreover, several clinical trials research the possibility of the therapeutic use of iPSC in combination with cytokine-based therapy (NCT03213964) and monoclonal antibodies (NCT03319459, NCT03841110).

#### 3.2.2. NK Cells and Cytokine-Based Therapy

A large block of research is aimed at studying the antitumor potential of NK cells via their stimulation with cytokines. IL-2 was one of the first NK cell-stimulating cytokines with proven efficacy in the treatment of solid tumors, particularly in metastatic melanoma and metastatic renal cancer [205]. However, how we describe the above life-threatening toxicities of IL-2 is the main reason that restricts the use of this cytokine in practice. Recently, Silva and colleagues have developed a mock analogue of IL-2 that binds to both CD122 and CD132 without binding to CD25 or IL-15Rα (CD215). This molecule, named Neo-2/15, induced increased activation of mouse CD4+ T cells and YT human NK cell line and higher therapeutic activity compared to conventional IL-2 in murine models of melanoma and colorectal cancer [206].

The therapeutic use of other NK cell-stimulated cytokines such as IL-12 and IL-18 to stimulate antibody-dependent cellular cytotoxicity or IL-15 and IL-21 to trigger NK cell proliferation, the increase in NK cell activating receptor expression, and cytokine production are now being studied against various types of solid tumors [207,208,209,210]. Preclinical studies showed IL-15 induced prolonged expansion and activation of NK cells, resulting in tumor regression, decreased metastasis, and increased survival in a mouse model of lung adenocarcinoma [211]. In the first-in-human phase I clinical trial, IL-15 administration led to increased proliferation and number of circulating NK cells in patients with metastatic malignant melanoma and metastatic renal cell cancer. Additionally, for two patients with lung cancer, the clearance of lung metastases was observed [167]. In another clinical trial, IL-15-stimulated NK cells infusion induced a clinical response after haplo-identical stem cell transplantation in four out of six pediatric patients with different solid refractory tumors [212]. Because of the main limitation in the use of IL-15 is its short half-life, generating a new agonistic form of IL-15 become one of the defined approaches to improve this situation. Recently created IL-15 superagonist ALT-803 has shown promising results in clinical studies for lymphoma [168] and NSCLC [213].

Several cytokines activating NK cells are also being evaluated for therapeutic potential. IL-12 and IL-18 are known as NK cell activating cytokines. It was demonstrated that combination of IL-12/IL-18/IL-15-preactivated NK cells persisted at high cell numbers with potent effector function in lymphoma and melanoma tumor models in human and mice [154]. IL-21 is another perspective cytokine, and its potential to induce antitumor response via activation of CD8 T cells, NK cells, and NKT cells has been shown in different studies [214,215]. Several preclinical trials demonstrated IL-21 antitumor activity in different cancer models [215,216,217]. Additionally, IL-21 efficacy was shown in phase I and II clinical trials in patients with renal carcinoma and metastatic melanomas, respectively [207,208]. However, the use of IL-21 is associated with side effects such as granulocytopenia and liver toxicity and colitis-associated colon cancer [218]. So, a deeper understanding of the biology of IL-21 is required to figure out its therapeutic potential.

#### 3.2.3. NK Cell-Directed Monoclonal Antibodies in Solid Tumors

Monoclonal antibody therapies for solid tumors are constantly evolving, but the problem is that a significant number of patients are not benefiting from this kind of therapy. This fact reflects novel strategic methods in the development of monoclonal antibody therapies. Optimization of molecular structures with high-affinity properties and different therapeutic combinations for antibodies are more actual approaches in this matter. It was shown that the combination of monalizumab (IgG4-blocking mAb against NKG2A) and anti-PD-L1 antibody provides enhanced NK cell effector functions and ADCC [52]. Monalizumab is being evaluated in a phase 1 clinical trial in combination with durvalumab (anti-PD-L1 mAb) in advanced solid tumors (NCT02671435). As we said above, the optimization of antibody molecular structure aimed to enhance therapeutic efficacy is an important area of the ongoing translational research. Umana et al. have created chimeric glycosylated anti-neuroblastoma IgG1 mAb (chCE7) increasing NK-cell mediated ADCC more than 20 fold [219]. Another approach to increase ADCC is to replace amino acids in the binding site of the gamma receptor fragment (FcγR). Lazar et al. have developed an Fc variant with an increased FcγRII/IIIa binding affinity due to amino acid replacement showing increased ability to induce NK-cell mediated ADCC compared to native IgG1 molecules [220]. In the past decade, accumulated knowledge on the biology of NK cell receptors has stimulated the development of bispecific mAbs aimed at increasing specificity and facilitating interactions between immune and tumor cells while reducing systemic toxicity. Since the creation of the first bispecific mAbs targeting CD16 on NK cells and CD30 on Hodgkin’s lymphoma cells more than two decades ago, bi- and trispecific mAbs have been designed to interact with erB2/CD16 in breast cancer [221], with EGFR being overexpressed in many different epithelial types of cancer [222], EPCAM in various cell carcinoma lines [223], and EPCAM/CD3 in serous endometrial cancer [224].

#### 3.2.4. Novel Combination Therapeutic Approaches for Treatment of Solid Tumors

A large number of clinical studies are being carried out using combination therapies, which include NK cell-based treatment. One of the most frequently used therapeutic drugs in the combination therapy of solid tumors are inhibitors of immune checkpoints (PD-1, PD-L1, CTLA-4, etc.). It has been shown in preclinical studies in a mouse model that the use of inhibitory antibodies to TIGIT can improve the antitumor effect of monoclonal antibodies against HER2-positive breast cancer cell lines either alone or in combination with PD1/PD-L1 inhibitors by increasing NK cell infiltration [225]. Other preclinical studies have shown that blocking CD96 in combination with anti-PD1 or anti-CTLA-4 enhanced NK cell infiltration and IFN-γ production, thereby reducing tumor metastases in the lungs for various types of solid tumors [226]. TIM-3 blockade enhanced cytotoxic function of NK cells against melanoma cells [227]. Therapeutic efficacy of the combined use of various immune checkpoint inhibitors such as anti-TIM-3 and anti-TIGIT is currently being tested in ongoing phase I and II clinical trials in patients with solid tumors (Table 1). In addition to immune checkpoints, another study is being conducted concerning the regulation of NK cell functional activity—by affecting the secretion of soluble ligands for NK-activating receptors by solid tumor cells, which have immunosuppressive effects. Tumor-derived soluble MHC I-chain-related molecule (sMIC) is highly immune suppressive and correlates with poor prognosis in cancer patients. In a mouse model, therapy with sMIC-neutralizing non-blocking anti-MIC mAb effectuated antitumor immune responses against various types of MIC (+) solid tumors [228]. In another similar study, the neutralization of soluble NKG2D ligands such as MICA and MICB with mAb B10G5 was effective against prostate cancer and its metastases, leading to increased infiltration with NK cells to tumor parenchyma and improving CTLA-4 blockade therapy [229]. More recently, Kamiya et al. have developed an approach to block NK cell inhibitory receptor NKG2A. These NK cells were characterized by increased cytotoxicity towards tumor cells expressing HLA-E [230]. Monalizumab (IPH2201) is a first-class anti-NKG2A blocking mAb currently undergoing several phase I/II clinical trials for safety and antitumor activity in various types of cancers, including chronic squamous cell carcinoma of the head and neck (SCCHN), gynecological cancers, and advanced colorectal cancer [52,231]. For advanced solid tumors, the combination of monalizumab and durvalumab (anti-PD-L1 mAb) has also been shown to be well tolerated without serious side effects. The preliminary results of a phase II clinical trial investigating the combination of monalizumab and cetuximab (anti-EGFR) in patients with SCCHN were quite encouraging, with an objective response rate of 31% [52].

Another approach to increasing NK-mediated killing of solid tumor cells is the use of low-dose chemotherapy. Several independent studies using cell lines and mouse models have shown that low-dose chemotherapy induced sensitivity of tumor cells to NK cell-mediated destruction in various types of solid tumors including kidney cancer, melanoma, colorectal cancer, and others via the activation of NKG2D or TRAIL ligand expression [232,233,234].

Currently, several phase I/II clinical trials are underway for the treatment of solid tumors based on the adoptive transfer of NK cells combined with other different cancer therapy types. In addition to the examples mentioned above, therapeutic components can include non-typical novel agents and methods, such as using cytokine-induced memory-like NK cells, immunomodulatory drugs, cryosurgery, and electroporation. The mAb lenalidomide should be highlighted among the most effective immunomodulatory drugs inducing surface ligand expression for NK-activating receptors in cancer cells [235]. It is undergoing clinical trials in combination therapy with adoptive NK cell transfer for patients with neuroblastoma (Table 1). It is likely that this approach can help to overcome the consequences of the tumor escape from the immune response [133,236].

## 4. Conclusions

The development of immune therapy has opened up new opportunities and prospects in the treatment of cancer. Such prospects seem promising, especially when it comes to new, but at the same time well-known, links of antitumor immune responses, including NK cell response. At present, a huge layer of research concerns studying the possibilities for the use of NK cells for the treatment of both hematological and solid tumors. Based on the examples of the various studies discussed above, it can be noted that most of the projects implemented in clinical practice and on the way to implementation are in the field of hematological tumor immunotherapy. In turn, for solid tumors, the number of clinical trials is still more modest, but the number of various preclinical trials is large. Such studies aim to find new strategies for solving problems of realizing the antitumor potential of NK cells in solid tumors. The main limitations of NK cells in solid tumors are associated with the complexity of migration of NK cells into the immediate tumor focus and probably with more highly developed TME than for hematological tumors. The number of studies continues to grow to this day. Thus, we can safely say that the prospects for development of NK cell therapy stretch far ahead, opening up new solutions for mankind on the way to confront cancer.

## Figures and Tables

**Table 1:** Clinical trials of NK cells for adoptive immunotherapy of solid tumors.

Adoptive Transfer of Expanded and Activated nk Cells				
NK sources	Combination	ClinicalTrial.gov number	Phase	Condition
Autologous	Comparison with allogenic	NCT02853903	II	Different solid tumors
	-	NCT03662477	Early I	Lung cancer
	Anti-PD-1 therapy	NCT03958097	I/II	Non-small cell lung cancer
	Anti-GD2 + lenalidomide	NCT02573896	I	Neuroblastoma
	Bortezomib	NCT00720785	I	Colorectal cancer, pancreatic cancer, non-small cell lung cancer
	Sintilimab	NCT03958097	II	Non-small cell lung cancer
Allogeneic	-	NCT04616209	I/II	Non-small cell lung cancer
	Comparison with autologous	NCT02853903	II	Different solid tumors
	Chemotherapy	NCT04162158	I/II	Hepatocellular carcinoma
	ALT-803	NCT02890758	I	Colorectal cancer, adenocarcinoma, soft tissue sarcoma, Ewing’s sarcoma, rhabdomyosarcoma
	Pemetrexed	NCT03366064	I	Non-small cell lung cancer
	Anti-GD2 + rIL2	NCT02650648	I	Neuroblastoma
	Pembrolizumab	NCT03937895	I/II	Biliary tract cancer
Not specified NK cells	Nivolumab	NCT02843204	I/II	Reccurent solid tumors
	Trastuzumab	NCT02843126	I/II	Breast cancer
	Cetuxirnab	NCT02845856	I/II	Non-small cell lung cancer
	Bevacizumab	NCT02857920	I/II	Reccurent metastatic solid tumors
	Irreversible electroporation	NCT02718859	I/II	Pancreatic cancer
		NCT03008343	I/II	Urothelial cancer
	Cryosurgery	NCT02844335	I/II	Breast cancer
		NCT02849314	I/II	Laryngeal cancer
		NCT02843802	I/II	Urothelial cancer
		NCT02849379	I/II	Tongue cancer
		NCT02849353	I/II	Ovarian cancer
		NCT02843607	I/II	Renal Cell carcinoma
		NCT02849366	I/II	Rhabdomyosarcoma
		NCT02843815	I/II	Non-small cell lung cancer
NK-92	ALT-803	NCT02465957	II	Merkel Cell carcinoma
UCB-derived	Chemotherapy	NCT03420963	I	Advanced solid tumors
		NCT03539406	I	Ovarian cancer
iPSC-derived	IL-2	NCT03213964	I	Ovarian cancer
	Anti-HER2 and anti-EGFR	NCT03319459	I	Advanced solid tumors
	Immune checkpoint inhibitors	NCT03841110	I	Advanced solid tumors
Cytokine-induced memory-like NK	IL-15 superagonist (N-803) and ipilimumab	NCT04290546	I	Squamous cell carcinoma
**Adoptive Transfer of car-nk Cells**				
CAR-NK	Drug combination	ClinicalTrial.gov number	Phase	Condition
ROB01 CAR-NK	-	NCT03940820	I/II	Different solid tumors
ROB01 BiCAR-NK	-	NCT03941457	I/II	Pancreatic cancer
ROB01 BiCAR-NK/T	-	NCT03931720	I/II	Malignant solid tumors
ErbB2/HER2 CAR-NK (NK-92/5.28z)	Intracranial injection	NCT03383978	I	Glioblastoma
CD-16A-IL2-NK-92 (haNK)	-	NCT03027128	I	Metastatic and Locally Advanced Solid tumors
	Chemotherapy, immune checkpoint inhibitors	NCT03387111	I/II	Squamous cell carcinoma
	IL-15 superagonist (N-803) and avelumab	NCT03853317	II	Mercel Cell carcinoma

## Data Availability

Not applicable.

## References

[1] Vivier E., Raulet D.H., Moretta A., Caligiuri M.A., Zitvogel L., Lanier L.L., Yokoyama W.M., Ugolini S. (2011). Innate or adaptive immunity? The example of natural killer cells. Science.

[2] Vivier E., Artis D., Colonna M., Diefenbach A., Di Santo J.P., Eberl G., Koyasu S., Locksley R.M., McKenzie A.N.J., Mebius R.E. (2018). Innate Lymphoid Cells: 10 Years On. Cell.

[3] Ljunggren H.G., Kärre K. (1990). In search of the “missing self”: MHC molecules and NK cell recognition. Immunol. Today.

[4] Vidal S.M., Khakoo S.I., Biron C.A. (2011). Natural killer cell responses during viral infections: Flexibility and conditioning of innate immunity by experience. Curr. Opin. Virol..

[5] Moretta L., Locatelli F., Pende D., Sivori S., Falco M., Bottino C., Mingari M.C., Moretta A. (2011). Human NK receptors: From the molecules to the therapy of high risk leukemias. FEBS Lett..

[6] Sun J.C., Lanier L.L. (2011). NK cell development, homeostasis and function: Parallels with CD8+ T cells. Nat. Rev. Immunol..

[7] Mizia-Malarz A., Sobol-Milejska G. (2019). NK cells as possible prognostic factor in childhood acute lymphoblastic leukemia. Dis. Markers.

[8] Sullivan E.M., Jeha S., Kang G., Cheng C., Rooney B., Holladay M., Bari R., Schell S., Tuggle M., Pui C.H. (2014). NK cell genotype and phenotype at diagnosis of acute lymphoblastic leukemia correlate with postinduction residual disease. Clin. Cancer Res..

[9] Palmer S., Hanson C.A., Zent C.S., Porrata L.F., LaPlant B., Geyer S.M., Markovic S.N., Call T.G., Bowen D.A., Jelinek D.F. (2008). Prognostic importance of T and NK-cells in a consecutive series of newly diagnosed patients with chronic lymphocytic leukaemia. Br. J. Haematol..

[10] Gonzalez-Rodriguez A.P., Contesti J., Huergo-Zapico L., Lopez-Soto A., Fernández-Guizn A., Acebes-Huerta A., Gonzalez-Huerta A.J., Gonzalez E., Fernandez-Alvarez C., Gonzalez S. (2010). Prognostic significance of CD8 and CD4 T cells in chronic lymphocytic leukemia. Leuk. Lymphoma.

[11] Plonquet A., Haioun C., Jais J.P., Debard A.L., Salles G., Bene M.C., Feugier P., Rabian C., Casasnovas O., Labalette M. (2007). Peripheral blood natural killer cell count is associated with clinical outcome in patients with aaIPI 2-3 diffuse large B-cell lymphoma. Ann. Oncol..

[12] Álvaro-Naranjo T., Lejeune M., Salvadó T., Príncep R.B., Reverter-Branchat G., Jaén-Martínez J., Pons-Ferré L.E. (2005). Tumor-infiltrating cells as a prognostic factor in Hodgkin’s lymphoma: A quantitative tissue microarray study in a large retrospective cohort of 267 patients. Leuk. Lymphoma.

[13] Szmania S., Lapteva N., Garg T., Greenway A., Lingo J., Nair B., Stone K., Woods E., Khan J., Stivers J. (2015). Ex vivo-expanded natural killer cells demonstrate robust proliferation in vivo in high-risk relapsed multiple myeloma patients. J. Immunother..

[14] Lee D.A. (2019). Cellular therapy: Adoptive immunotherapy with expanded natural killer cells. Immunol. Rev..

[15] Al-Anazi K., Al-Jasser A., Al-Anazi W. (2019). Natural killer cells in patients with hematologic malignancies, solid tumors and in recipients of hematopoietic stem cell transplantation. J. Stem Cell Ther. Transplant..

[16] Blom B., van Hoeven V., Hazenberg M.D. (2019). ILCs in hematologic malignancies: Tumor cell killers and tissue healers. Semin. Immunol..

[17] Epling-Burnette P.K., Bai F., Painter J.S., Rollison D.E., Salih H.R., Krusch M., Zou J.X., Ku E., Zhong B., Boulware D. (2007). Reduced natural killer (NK) function associated with high-risk myelodysplastic syndrome (MDS) and reduced expression of activating NK receptors. Blood.

[18] Mace E.M., Orange J.S. (2016). Genetic causes of human NK cell deficiency and their effect on NK cell subsets. Front. Immunol..

[19] Eidenschenk C., Dunne J., Jouanguy E., Fourlinnie C., Gineau L., Bacq D., McMahon C., Smith O., Casanova J.L., Abel L. (2006). A novel primary immunodeficiency with specific natural-killer cell deficiency maps to the centromeric region of chromosome 8. Am. J. Hum. Genet..

[20] Cannella S., Santoro A., Bruno G., Pillon M., Mussolin L., Mangili G., Rosolen A., Aricò M. (2007). Germline mutations of the perforin gene are a frequent occurrence in childhood anaplastic large cell lymphoma. Cancer.

[21] Yang L., Liu H., Zhao J., Da W., Zheng J., Wang L., Li G., Zhu P. (2011). Mutations of perforin gene in Chinese patients with acute lymphoblastic leukemia. Leuk. Res..

[22] Clementi R., Locatelli F., Dupré L., Garaventa A., Emmi L., Bregni M., Cefalo G., Moretta A., Danesino C., Comis M. (2005). A proportion of patients with lymphoma may harbor mutations of the perform gene. Blood.

[23] Correia D.V., Fogli M., Hudspeth K., Da Silva M.G., Mavilio D., Silva-Santos B. (2011). Differentiation of human peripheral blood Vδ1+ T cells expressing the natural cytotoxicity receptor NKp30 for recognition of lymphoid leukemia cells. Blood.

[24] Martner A., Rydström A., Riise R.E., Aurelius J., Brune M., Foà R., Hellstrand K., Thorén F.B. (2015). NK cell expression of natural cytotoxicity receptors may determine relapse risk in older AML patients undergoing immunotherapy for remission maintenance. Oncotarget.

[25] Pende D., Spaggiari G.M., Marcenaro S., Martini S., Rivera P., Capobianco A., Falco M., Lanino E., Pierri I., Zambello R. (2005). Analysis of the receptor-ligand interactions in the natural killer-mediated lysis of freshly isolated myeloid or lymphoblastic leukemias: Evidence for the involvement of the Polio virus receptor (CD 155) and Nectin-2 (CD 112). Blood.

[26] Lion E., Willemen Y., Berneman Z.N., Van Tendeloo V.F.I., Smits E.L.J. (2012). Natural killer cell immune escape in acute myeloid leukemia. Leukemia.

[27] Costello R.T., Knoblauch B., Sanchez C., Mercier D., Le Treut T., Sébahoun G. (2012). Expression of natural killer cell activating receptors in patients with chronic lymphocytic leukaemia. Immunology.

[28] Fauriat C., Just-Landi S., Mallet F., Arnoulet C., Sainty D., Olive D., Costello R.T. (2007). Deficient expression of NCR in NK cells from acute myeloid leukemia: Evolution during leukemia treatment and impact of leukemia cells in NCR dull phenotype induction. Blood.

[29] Della Chiesa M., Carlomagno S., Frumento G., Balsamo M., Cantoni C., Conte R., Moretta L., Moretta A., Vitale M. (2006). The tryptophan catabolite l-kynurenine inhibits the surface expression of NKp46- and NKG2D-activating receptors and regulates NK-cell function. Blood.

[30] Li H., Han Y., Guo Q., Zhang M., Cao X. (2009). Cancer-Expanded Myeloid-Derived Suppressor Cells Induce Anergy of NK Cells through Membrane-Bound TGF-β1. J. Immunol..

[31] Barrow A.D., Martin C.J., Colonna M. (2019). The natural cytotoxicity receptors in health and disease. Front. Immunol..

[32] Vitale M., Cantoni C., Della Chiesa M., Ferlazzo G., Carlomagno S., Pende D., Falco M., Pessino A., Muccio L., De Maria A. (2019). An historical overview: The discovery of how NK cells can kill enemies, recruit defense troops, and more. Front. Immunol..

[33] Lam R.A., Chwee J.Y., Le Bert N., Sauer M., Pogge Von Strandmann E., Gasser S. (2013). Regulation of self-ligands for activating natural killer cell receptors. Ann. Med..

[34] Schlecker E., Fiegler N., Arnold A., Altevogt P., Rose-John S., Moldenhauer G., Sucker A., Paschen A., Von Strandmann E.P., Textor S. (2014). Metalloprotease-mediated tumor cell shedding of B7-H6, the ligand of the natural killer cell-activating receptor NKp30. Cancer Res..

[35] Pesce S., Tabellini G., Cantoni C., Patrizi O., Coltrini D., Rampinelli F., Matta J., Vivier E., Moretta A., Parolini S. (2015). B7-H6-mediated downregulation of NKp30 in NK cells contributes to ovarian carcinoma immune escape. Oncoimmunology.

[36] Gaggero S., Bruschi M., Petretto A., Parodi M., Del Zotto G., Lavarello C., Prato C., Santucci L., Barbuto A., Bottino C. (2018). Nidogen-1 is a novel extracellular ligand for the NKp44 activating receptor. Oncoimmunology.

[37] Reiners K.S., Topolar D., Henke A., Simhadri V.R., Kessler J., Sauer M., Bessler M., Hansen H.P., Tawadros S., Herling M. (2013). Soluble ligands for NK cell receptors promote evasion of chronic lymphocytic leukemia cells from NK cell anti-tumor activity. Blood.

[38] Raulet D.H., Gasser S., Gowen B.G., Deng W., Jung H. (2013). Regulation of ligands for the NKG2D activating receptor. Annu. Rev. Immunol..

[39] Hilpert J., Grosse-Hovest L., Grünebach F., Buechele C., Nuebling T., Raum T., Steinle A., Salih H.R. (2012). Comprehensive Analysis of NKG2D Ligand Expression and Release in Leukemia: Implications for NKG2D-Mediated NK Cell Responses. J. Immunol..

[40] Nückel H., Switala M., Sellmann L., Horn P.A., Dürig J., Dührsen U., Küppers R., Grosse-Wilde H., Rebmann V. (2010). The prognostic significance of soluble NKG2D ligands in B-cell chronic lymphocytic leukemia. Leukemia.

[41] Huergo-Zapico L., Acebes-Huerta A., Gonzalez-Rodriguez A.P., Contesti J., Gonzalez-Garcia E., Payer A.R., Villa-Alvarez M., Fernández-Guizán A., López-Soto A., Gonzalez S. (2014). Expansion of NK cells and reduction of NKG2D expression in chronic lymphocytic leukemia. Correlation with progressive disease. PLoS ONE.

[42] Deng W., Gowen B.G., Zhang L., Wang L., Lau S., Iannello A., Xu J., Rovis T.L., Xiong N., Raulet D.H. (2015). A shed NKG2D ligand that promotes natural killer cell activation and tumor rejection. Science.

[43] Chiossone L., Vienne M., Kerdiles Y.M., Vivier E. (2017). Natural killer cell immunotherapies against cancer: Checkpoint inhibitors and more. Semin. Immunol..

[44] Muntasell A., Ochoa M.C., Cordeiro L., Berraondo P., de Cerio A.L.-D., Cabo M., López-Botet M., Melero I. (2017). Targeting NK-cell checkpoints for cancer immunotherapy. Curr. Opin. Immunol..

[45] Hobo W., Hutten T.J.A., Schaap N.P.M., Dolstra H. (2018). Immune checkpoint molecules in acute myeloid leukaemia: Managing the double-edged sword. Br. J. Haematol..

[46] Benson D.M., Bakan C.E., Mishra A., Hofmeister C.C., Efebera Y., Becknell B., Baiocchi R.A., Zhang J., Yu J., Smith M.K. (2010). The PD-1/PD-L1 axis modulates the natural killer cell versus multiple myeloma effect: A therapeutic target for CT-011, a novel monoclonal anti-PD-1 antibody. Blood.

[47] Giuliani M., Janji B., Berchem G. (2017). Activation of NK cells and disruption of PD-L1/PD-1 axis: Two different ways for lenalidomide to block myeloma progression. Oncotarget.

[48] Hsu J., Hodgins J.J., Marathe M., Nicolai C.J., Bourgeois-Daigneault M.C., Trevino T.N., Azimi C.S., Scheer A.K., Randolph H.E., Thompson T.W. (2018). Contribution of NK cells to immunotherapy mediated by PD-1/PD-L1 blockade. J. Clin. Investig..

[49] Vari F., Arpon D., Keane C., Hertzberg M.S., Talaulikar D., Jain S., Cui Q., Han E., Tobin J., Bird R. (2018). Immune evasion via PD-1/PD-L1 on NK cells and monocyte/macrophages is more prominent in Hodgkin lymphoma than DLBCL. Blood.

[50] Hadadi L., Hafezi M., Amirzargar A.A., Sharifian R.A., Abediankenari S., Asgarian-Omran H. (2019). Dysregulated Expression of Tim-3 and NKp30 Receptors on NK Cells of Patients with Chronic Lymphocytic Leukemia. Oncol. Res. Treat..

[51] Zhang Q., Bi J., Zheng X., Chen Y., Wang H., Wu W., Wang Z., Wu Q., Peng H., Wei H. (2018). Blockade of the checkpoint receptor TIGIT prevents NK cell exhaustion and elicits potent anti-tumor immunity. Nat. Immunol..

[52] André P., Denis C., Soulas C., Bourbon-Caillet C., Lopez J., Arnoux T., Bléry M., Bonnafous C., Gauthier L., Morel A. (2018). Anti-NKG2A mAb Is a Checkpoint Inhibitor that Promotes Anti-tumor Immunity by Unleashing Both T and NK Cells. Cell.

[53] Van Montfoort N., Borst L., Korrer M.J., Sluijter M., Marijt K.A., Santegoets S.J., van Ham V.J., Ehsan I., Charoentong P., André P. (2018). NKG2A blockade potentiates CD8 T-cell immunity induced by cancer vaccines. Cell.

[54] Jurisic V., Srdic T., Konjevic G., Markovic O., Colovic M. (2007). Clinical stage-depending decrease of NK cell activity in multiple myeloma patients. Med. Oncol..

[55] García-Sanz R., González M., Orfao A., Moro M.J., Hernández J.M., Borrego D., Carnero M., Casanova F., Bárez A., Jiménez R. (1996). Analysis of natural killer-associated antigens in peripheral blood and bone marrow of multiple myeloma patients and prognostic implications. Br. J. Haematol..

[56] Mundy-Bosse B., Denlinger N., McLaughlin E., Chakravarti N., Hwang S., Chen L., Mao H.C., Kline D., Youssef Y., Kohnken R. (2018). Highly cytotoxic natural killer cells are associated with poor prognosis in patients with cutaneous T-cell lymphoma. Blood Adv..

[57] San Miguel J.F., García-Sanz R. (2005). Prognostic features of multiple myeloma. Best Pract. Res. Clin. Haematol..

[58] Malmberg K.J., Carlsten M., Björklund A., Sohlberg E., Bryceson Y.T., Ljunggren H.G. (2017). Natural killer cell-mediated immunosurveillance of human cancer. Semin. Immunol..

[59] Spiegel A., Brooks M.W., Houshyar S., Reinhardt F., Ardolino M., Fessler E., Chen M.B., Krall J.A., Decock J., Zervantonakis I.K. (2016). Neutrophils suppress intraluminal NK cell-mediated tumor cell clearance and enhance extravasation of disseminated carcinoma cells. Cancer Discov..

[60] Malladi S., Macalinao D.G., Jin X., He L., Basnet H., Zou Y., de Stanchina E., Massagué J. (2016). Metastatic Latency and Immune Evasion Through Autocrine Inhibition of WNT. Cell.

[61] Glasner A., Ghadially H., Gur C., Stanietsky N., Tsukerman P., Enk J., Mandelboim O. (2012). Recognition and prevention of tumor metastasis by the NK receptor NKp46/NCR1. J. Immunol..

[62] Iguchi-Manaka A., Kai H., Yamashita Y., Shibata K., Tahara-Hanaoka S., Honda S.-I., Yasui T., Kikutani H., Shibuya K., Shibuya A. (2008). Accelerated tumor growth in mice deficient in DNAM-1 receptor. J. Exp. Med..

[63] Tartter P.I., Steinberg B., Barron D.M., Martinelli G. (1987). The prognostic significance of natural killer cytotoxicity in patients with colorectal cancer. Arch. Surg..

[64] Liljefors M.G., Nilsson B., Skog A.-L.H., Ragnhammar P., Mellstedt H., Frödin J.-E. (2003). Natural killer (NK) cell function is a strong prognostic factor in colorectal carcinoma patients treated with the monoclonal antibody 17-1A. Int. J. Cancer.

[65] Coppola A., Arriga R., Lauro D., del Principe M.I., Buccisano F., Maurillo L., Palomba P., Venditti A., Sconocchia G. (2015). NK Cell Inflammation in the Clinical Outcome of Colorectal Carcinoma. Front. Med..

[66] Gulubova M., Manolova I., Kyurkchiev D., Julianov A., Altunkova I. (2009). Decrease in intrahepatic CD56+ lymphocytes in gastric and colorectal cancer patients with liver metastases. Apmis.

[67] Maréchal R., De Schutter J., Nagy N., Demetter P., Lemmers A., Devière J., Salmon I., Tejpar S., Van Laethem J.-L. (2010). Putative contribution of CD56 positive cells in cetuximab treatment efficacy in first-line metastatic colorectal cancer patients. BMC Cancer.

[68] Halama N., Braun M., Kahlert C., Spille A., Quack C., Rahbari N., Koch M., Weitz J., Kloor M., Zoernig I. (2011). Natural killer cells are scarce in colorectal carcinoma tissue despite high levels of chemokines and cytokines. Clin. Cancer Res..

[69] Ali T.H., Pisanti S., Ciaglia E., Mortarini R., Anichini A., Garofalo C., Tallerico R., Santinami M., Gulletta E., Ietto C. (2014). Enrichment of CD56dimKIR+CD57+ highly cytotoxic NK cells in tumour-infiltrated lymph nodes of melanoma patients. Nat. Commun..

[70] Messaoudene M., Fregni G., Fourmentraux-Neves E., Chanal J., Maubec E., Mazouz-Dorval S., Couturaud B., Girod A., Sastre-Garau X., Albert S. (2014). Mature cytotoxic CD56bright/CD16+ natural killer cells can infiltrate lymph nodes adjacent to metastatic melanoma. Cancer Res..

[71] Denkert C., Von Minckwitz G., Darb-Esfahani S., Lederer B., Heppner B.I., Weber K.E., Budczies J., Huober J., Klauschen F., Furlanetto J. (2018). Tumour-infiltrating lymphocytes and prognosis in different subtypes of breast cancer: A pooled analysis of 3771 patients treated with neoadjuvant therapy. Lancet Oncol..

[72] Pernot S., Terme M., Radosevic-Robin N., Castan F., Badoual C., Marcheteau E., Penault-Llorca F., Bouche O., Bennouna J., Francois E. (2020). Infiltrating and peripheral immune cell analysis in advanced gastric cancer according to the Lauren classification and its prognostic significance. Gastric Cancer.

[73] Ménard C., Blay J.Y., Borg C., Michiels S., Ghiringhelli F., Robert C., Nonn C., Chaput N., Taïeb J., Delahaye N.F. (2009). Natural killer cell IFN-γ levels predict long-term survival with imatinib mesylate therapy in gastrointestinal stromal tumor-bearing patients. Cancer Res..

[74] Semeraro M., Rusakiewicz S., Minard-Colin V., Delahaye N.F., Enot D., Vély F., Marabelle A., Papoular B., Piperoglou C., Ponzoni M. (2015). Clinical impact of the NKp30/B7-H6 axis in high-risk neuroblastoma patients. Sci. Transl. Med..

[75] Zhu L.-Y., Zhou J., Liu Y.-Z., Pan W.-D. (2009). Prognostic significance of natural killer cell infiltration in hepatocellular carcinoma. Ai Zheng.

[76] Kmiecik J., Zimmer J., Chekenya M. (2014). Natural killer cells in intracranial neoplasms: Presence and therapeutic efficacy against brain tumours. J. Neurooncol..

[77] Habif G., Crinier A., André P., Vivier E., Narni-Mancinelli E. (2019). Targeting natural killer cells in solid tumors. Cell. Mol. Immunol..

[78] Pasero C., Gravis G., Guerin M., Granjeaud S., Thomassin-Piana J., Rocchi P., Paciencia-Gros M., Poizat F., Bentobji M., Azario-Cheillan F. (2016). Inherent and tumor-driven immune tolerance in the prostate microenvironment impairs natural killer cell antitumor activity. Cancer Res..

[79] Garcia-Iglesias T., del Toro-Arreola A., Albarran-Somoza B., del Toro-Arreola S., Sanchez-Hernandez P.E., Ramirez-Dueñas M., Balderas-Peña L.M.A., Bravo-Cuellar A., Ortiz-Lazareno P.C., Daneri-Navarro A. (2009). Low NKp30, NKp46 and NKG2D expression and reduced cytotoxic activity on NK cells in cervical cancer and precursor lesions. BMC Cancer.

[80] Rusakiewicz S., Perier A., Semeraro M., Pitt J.M., von Strandmann E.P., Reiners K.S., Aspeslagh S., Pipéroglou C., Vély F., Ivagnes A. (2017). NKp30 isoforms and NKp30 ligands are predictive biomarkers of response to imatinib mesylate in metastatic GIST patients. Oncoimmunology.

[81] Mantovani S., Oliviero B., Lombardi A., Varchetta S., Mele D., Sangiovanni A., Rossi G., Donadon M., Torzilli G., Soldani C. (2019). Deficient Natural Killer Cell NKp30-Mediated Function and Altered NCR3 Splice Variants in Hepatocellular Carcinoma. Hepatology.

[82] Barrow A.D., Edeling M.A., Trifonov V., Luo J., Goyal P., Bohl B., Bando J., Kim A.H., Walker J., Andahazy M. (2018). Natural Killer cells control tumor growth by sensing a growth factor. Cell.

[83] Li L., Zhang Y., Li N., Feng L., Yao H., Zhang R., Li B., Li X., Han N., Gao Y. (2015). Nidogen-1: A candidate biomarker for ovarian serous cancer. Jpn. J. Clin. Oncol..

[84] Willumsen N., Bager C.L., Leeming D.J., Bay-Jensen A.C., Karsdal M.A. (2017). Nidogen-1 Degraded by Cathepsin S can be Quantified in Serum and is Associated with Non–Small Cell Lung Cancer. Neoplasia.

[85] Sun Y., Sedgwick A.J., Palarasah Y., Mangiola S., Barrow A.D. (2021). A Transcriptional Signature of PDGF-DD Activated Natural Killer Cells Predicts More Favorable Prognosis in Low-Grade Glioma. Front. Immunol..

[86] Schleypen J.S., Baur N., Kammerer R., Nelson P.J., Rohrmann K., Gröne E.F., Hohenfellner M., Haferkamp A., Pohla H., Schendel D.J. (2006). Cytotoxic markers and frequency predict functional capacity of natural killer cells infiltrating renal cell carcinoma. Clin. Cancer Res..

[87] Wu Y., Kuang D.M., Pan W.D., Wan Y.-L., Lao X.M., Wang D., Li X.F., Zheng L. (2013). Monocyte/macrophage-elicited natural killer cell dysfunction in hepatocellular carcinoma is mediated by CD48/2B4 interactions. Hepatology.

[88] Schleypen J.S., Von Geldern M., Weiß E.H., Kotzias N., Rohrmann K., Schendel D.J., Falk C.S., Pohla H. (2003). Renal cell carcinoma-infiltrating natural killer cells express differential repertoires of activating and inhibitory receptors and are inhibited by specific HLA class I allotypes. Int. J. Cancer.

[89] Delahaye N.F., Rusakiewicz S., Martins I., Ménard C., Roux S., Lyonnet L., Paul P., Sarabi M., Chaput N., Semeraro M. (2011). Alternatively spliced NKp30 isoforms affect the prognosis of gastrointestinal stromal tumors. Nat. Med..

[90] Li T., Yang Y., Hua X., Wang G., Liu W., Jia C., Tai Y., Zhang Q., Chen G. (2012). Hepatocellular carcinoma-associated fibroblasts trigger NK cell dysfunction via PGE2 and IDO. Cancer Lett..

[91] Lavin Y., Kobayashi S., Leader A., Amir E.-A.D., Elefant N., Bigenwald C., Remark R., Sweeney R., Becker C.D., Levine J.H. (2017). Innate Immune Landscape in Early Lung Adenocarcinoma by Paired Single-Cell Analyses. Cell.

[92] Platonova S., Cherfils-Vicini J., Damotte D., Crozet L., Vieillard V., Validire P., André P., Dieu-Nosjean M.C., Alifano M., Régnard J.F. (2011). Profound coordinated alterations of intratumoral NK cell phenotype and function in lung carcinoma. Cancer Res..

[93] Esendagli G., Bruderek K., Goldmann T., Busche A., Branscheid D., Vollmer E., Brandau S. (2008). Malignant and non-malignant lung tissue areas are differentially populated by natural killer cells and regulatory T cells in non-small cell lung cancer. Lung Cancer.

[94] Carrega P., Morandi B., Costa R., Frumento G., Forte G., Altavilla G., Ratto G.B., Mingari M.C., Moretta L., Ferlazzo G. (2008). Natural killer cells infiltrating human nonsmall-cell lung cancer are enriched in CD56brightCD16- cells and display an impaired capability to kill tumor cells. Cancer.

[95] Russick J., Joubert P.E., Gillard-Bocquet M., Torset C., Meylan M., Petitprez F., Dragon-Durey M.A., Marmier S., Varthaman A., Josseaume N. (2020). Natural killer cells in the human lung tumor microenvironment display immune inhibitory functions. J. Immunother. Cancer.

[96] Putz E.M., Mayfosh A.J., Kos K., Barkauskas D.S., Nakamura K., Town L., Goodall K.J., Yee D.Y., Poon I.K.H., Baschuk N. (2017). NK cell heparanase controls tumor invasion and immune surveillance. J. Clin. Investig..

[97] Belli C., Trapani D., Viale G., D’Amico P., Duso B.A., Della Vigna P., Orsi F., Curigliano G. (2018). Targeting the microenvironment in solid tumors. Cancer Treat. Rev..

[98] Castriconi R., Dondero A., Bellora F., Moretta L., Castellano A., Locatelli F., Corrias M.V., Moretta A., Bottino C. (2013). Neuroblastoma-Derived TGF-β1 Modulates the Chemokine Receptor Repertoire of Human Resting NK Cells. J. Immunol..

[99] Lee J.-C., Lee K.-M., Kim D.-W., Heo D.S. (2004). Elevated TGF-β1 Secretion and Down-Modulation of NKG2D Underlies Impaired NK Cytotoxicity in Cancer Patients. J. Immunol..

[100] Holmgaard R.B., Schaer D.A., Li Y., Castaneda S.P., Murphy M.Y., Xu X., Inigo I., Dobkin J., Manro J.R., Iversen P.W. (2018). Targeting the TGFβ pathway with galunisertib, a TGFβRI small molecule inhibitor, promotes anti-tumor immunity leading to durable, complete responses, as monotherapy and in combination with checkpoint blockade. J. Immunother. Cancer.

[101] Mariathasan S., Turley S.J., Nickles D., Castiglioni A., Yuen K., Wang Y., Kadel E.E., Koeppen H., Astarita J.L., Cubas R. (2018). TGFβ attenuates tumour response to PD-L1 blockade by contributing to exclusion of T cells. Nature.

[102] O’Brien K.L., Finlay D.K. (2019). Immunometabolism and natural killer cell responses. Nat. Rev. Immunol..

[103] Cong J., Wang X., Zheng X., Wang D., Fu B., Sun R., Tian Z., Wei H. (2018). Dysfunction of Natural Killer Cells by FBP1-Induced Inhibition of Glycolysis during Lung Cancer Progression. Cell Metab..

[104] Assmann N., O’Brien K.L., Donnelly R.P., Dyck L., Zaiatz-Bittencourt V., Loftus R.M., Heinrich P., Oefner P.J., Lynch L., Gardiner C.M. (2017). Srebp-controlled glucose metabolism is essential for NK cell functional responses. Nat. Immunol..

[105] Li D., Long W., Huang R., Chen Y., Xia M. (2018). 27-Hydroxycholesterol Inhibits Sterol Regulatory Element-Binding Protein 1 Activation and Hepatic Lipid Accumulation in Mice. Obesity.

[106] Adams C.M., Reitz J., De Brabander J.K., Feramisco J.D., Li L., Brown M.S., Goldstein J.L. (2004). Cholesterol and 25-hydroxycholesterol inhibit activation of SREBPs by different mechanisms, both involving SCAP and insigs. J. Biol. Chem..

[107] Michelet X., Dyck L., Hogan A., Loftus R.M., Duquette D., Wei K., Beyaz S., Tavakkoli A., Foley C., Donnelly R. (2018). Metabolic reprogramming of natural killer cells in obesity limits antitumor responses. Nat. Immunol..

[108] Ma X., Holt D., Kundu N., Reader J., Goloubeva O., Take Y., Fulton A.M. (2013). A prostaglandin E (PGE) receptor EP4 antagonist protects natural killer cells from PGE2-mediated immunosuppression and inhibits breast cancer metastasis. Oncoimmunology.

[109] Li T., Zhang Q., Jiang Y., Yu J., Hu Y., Mou T., Chen G., Li G. (2016). Gastric cancer cells inhibit natural killer cell proliferation and induce apoptosis via prostaglandin E2. Oncoimmunology.

[110] Gubbels J.A.A., Felder M., Horibata S., Belisle J.A., Kapur A., Holden H., Petrie S., Migneault M., Rancourt C., Connor J.P. (2010). MUC16 provides immune protection by inhibiting synapse formation between NK and ovarian tumor cells. Mol. Cancer.

[111] Balsamo M., Vermi W., Parodi M., Pietra G., Manzini C., Queirolo P., Lonardi S., Augugliaro R., Moretta A., Facchetti F. (2012). Melanoma cells become resistant to NK-cell-mediated killing when exposed to NK-cell numbers compatible with NK-cell infiltration in the tumor. Eur. J. Immunol..

[112] Horton N.C., Mathew S.O., Mathew P.A. (2013). Novel Interaction between Proliferating Cell Nuclear Antigen and HLA I on the Surface of Tumor Cells Inhibits NK Cell Function through NKp44. PLoS ONE.

[113] Rosental B., Brusilovsky M., Hadad U., Oz D., Appel M.Y., Afergan F., Yossef R., Rosenberg L.A., Aharoni A., Cerwenka A. (2011). Proliferating Cell Nuclear Antigen Is a Novel Inhibitory Ligand for the Natural Cytotoxicity Receptor NKp44. J. Immunol..

[114] Melaiu O., Lucarini V., Cifaldi L., Fruci D. (2020). Influence of the Tumor Microenvironment on NK Cell Function in Solid Tumors. Front. Immunol..

[115] Beldi-Ferchiou A., Caillat-Zucman S. (2017). Control of NK cell activation by immune checkpoint molecules. Int. J. Mol. Sci..

[116] Kim N., Kim H.S. (2018). Targeting checkpoint receptors and molecules for therapeutic modulation of natural killer cells. Front. Immunol..

[117] Molgora M., Bonavita E., Ponzetta A., Riva F., Barbagallo M., Jaillon S., Popović B., Bernardini G., Magrini E., Gianni F. (2017). IL-1R8 is a checkpoint in NK cells regulating anti-tumour and anti-viral activity. Nature.

[118] Handgretinger R. (2012). New approaches to graft engineering for haploidentical bone marrow transplantation. Semin. Oncol..

[119] Reisner Y., Hagin D., Martelli M.F. (2011). Haploidentical hematopoietic transplantation: Current status and future perspectives. Blood.

[120] Locatelli F., Pende D., Falco M., Della Chiesa M., Moretta A., Moretta L. (2018). NK Cells Mediate a Crucial Graft-versus-Leukemia Effect in Haploidentical-HSCT to Cure High-Risk Acute Leukemia. Trends Immunol..

[121] Handgretinger R., Lang P., André M.C. (2016). Exploitation of natural killer cells for the treatment of acute leukemia. Blood.

[122] Cooley S., Parham P., Miller J.S. (2018). Strategies to activate NK cells to prevent relapse and induce remission following hematopoietic stem cell transplantation. Blood.

[123] Mehta R.S., Rezvani K. (2016). Immune reconstitution post allogeneic transplant and the impact of immune recovery on the risk of infection. Virulence.

[124] Anfossi N., André P., Guia S., Falk C.S., Roetynck S., Stewart C.A., Breso V., Frassati C., Reviron D., Middleton D. (2006). Human NK Cell Education by Inhibitory Receptors for MHC Class I. Immunity.

[125] Ruggeri L., Capanni M., Urbani E., Perruccio K., Shlomchik W.D., Tosti A., Posati S., Rogaia D., Frassoni F., Aversa F. (2002). Effectiveness of donor natural killer cell aloreactivity in mismatched hematopoietic transplants. Science.

[126] Bethge W.A., Haegele M., Faul C., Lang P., Schumm M., Bornhauser M., Handgretinger R., Kanz L. (2006). Haploidentical allogeneic hematopoietic cell transplantation in adults with reduced-intensity conditioning and CD3/CD19 depletion: Fast engraftment and low toxicity. Exp. Hematol..

[127] Lang P., Teltschik H.M., Feuchtinger T., Müller I., Pfeiffer M., Schumm M., Ebinger M., Schwarze C.P., Gruhn B., Schrauder A. (2014). Transplantation of CD3/CD19 depleted allografts from haploidentical family donors in paediatric leukaemia. Br. J. Haematol..

[128] Mehta R.S., Randolph B., Daher M., Rezvani K. (2018). NK cell therapy for hematologic malignancies. Int. J. Hematol..

[129] Brunstein C.G., Wagner J.E., Weisdorf D.J., Cooley S., Noreen H., Barker J.N., DeFor T., Verneris M.R., Blazar B.R., Miller J.S. (2009). Negative effect of KIR alloreactivity in recipients of umbilical cord blood transplant depends on transplantation conditioning intensity. Blood.

[130] Rocha V., Ruggeri A., Spellman S., Wang T., Sobecks R., Locatelli F., Askar M., Michel G., Arcese W., Iori A.P. (2016). Killer Cell Immunoglobulin-Like Receptor-Ligand Matching and Outcomes after Unrelated Cord Blood Transplantation in Acute Myeloid Leukemia. Biol. Blood Marrow Transplant..

[131] Cooley S., He F., Bachanova V., Vercellotti G.M., DeFor T.E., Curtsinger J.M., Robertson P., Grzywacz B., Conlon K.C., Waldmann T.A. (2019). First-in-human trial of rhIL-15 and haploidentical natural killer cell therapy for advanced acute myeloid leukemia. Blood Adv..

[132] Fehniger T.A., Miller J.S., Stuart R.K., Cooley S., Salhotra A., Curtsinger J., Westervelt P., DiPersio J.F., Hillman T.M., Silver N. (2018). A Phase 1 Trial of CNDO-109–Activated Natural Killer Cells in Patients with High-Risk Acute Myeloid Leukemia. Biol. Blood Marrow Transplant..

[133] Veluchamy J.P., Kok N., van der Vliet H.J., Verheul H.M.W., de Gruijl T.D., Spanholtz J. (2017). The rise of allogeneic Natural killer cells as a platform for cancer immunotherapy: Recent innovations and future developments. Front. Immunol..

[134] Van Elssen C.H.M.J., Ciurea S.O. (2018). NK cell therapy after hematopoietic stem cell transplantation: Can we improve anti-tumor effect?. Int. J. Hematol..

[135] Lupo K.B., Matosevic S. (2019). Natural killer cells as allogeneic effectors in adoptive cancer immunotherapy. Cancers.

[136] Killig M., Friedrichs B., Meisig J., Gentilini C., Blüthgen N., Loddenkemper C., Labopin M., Basara N., Pfrepper C., Niederwieser D.W. (2014). Tracking in vivo dynamics of NK cells transferred in patients undergoing stem cell transplantation. Eur. J. Immunol..

[137] Gill S., June C.H. (2015). Going viral: Chimeric antigen receptor T-cell therapy for hematological malignancies. Immunol. Rev..

[138] Maude S.L., Laetsch T.W., Buechner J., Rives S., Boyer M., Bittencourt H., Bader P., Verneris M.R., Stefanski H.E., Myers G.D. (2018). Tisagenlecleucel in Children and Young Adults with B-Cell Lymphoblastic Leukemia. N. Engl. J. Med..

[139] Neelapu S.S., Locke F.L., Bartlett N.L., Lekakis L.J., Miklos D.B., Jacobson C.A., Braunschweig I., Oluwole O.O., Siddiqi T., Lin Y. (2017). Axicabtagene Ciloleucel CAR T-Cell Therapy in Refractory Large B-Cell Lymphoma. N. Engl. J. Med..

[140] Imai C., Iwamoto S., Campana D. (2005). Genetic modification of primary natural killer cells overcomes inhibitory signals and induces specific killing of leukemic cells. Blood.

[141] Müller T., Uherek C., Maki G., Chow K.U., Schimpf A., Klingemann H.G., Tonn T., Wels W.S. (2008). Expression of a CD20-specific chimeric antigen receptor enhances cytotoxic activity of NK cells and overcomes NK-resistance of lymphoma and leukemia cells. Cancer Immunol. Immunother..

[142] Kloess S., Kretschmer A., Stahl L., Fricke S., Koehl U. (2019). CAR-Expressing Natural Killer Cells for Cancer Retargeting. Transfus. Med. Hemother..

[143] You F., Wang Y., Jiang L., Zhu X., Chen D., Yuan L., An G., Meng H., Yang L. (2019). A novel CD7 chimeric antigen receptor-modified NK-92MI cell line targeting T-cell acute lymphoblastic leukemia. Am. J. Cancer Res..

[144] Chen K.H., Wada M., Firor A.E., Pinz K.G., Jares A., Liu H., Salman H., Golightly M., Lan F., Jiang X. (2016). Novel anti-CD3 chimeric antigen receptor targeting of aggressive T cell malignancies. Oncotarget.

[145] Chen K.H., Wada M., Pinz K.G., Liu H., Lin K.W., Jares A., Firor A.E., Shuai X., Salman H., Golightly M. (2017). Preclinical targeting of aggressive T-cell malignancies using anti-CD5 chimeric antigen receptor. Leukemia.

[146] Tang X., Yang L., Li Z., Nalin A.P., Dai H., Xu T., Yin J., You F., Zhu M., Shen W. (2018). First-in-man clinical trial of CAR NK-92 cells: Safety test of CD33-CAR NK-92 cells in patients with relapsed and refractory acute myeloid leukemia. Am. J. Cancer Res..

[147] Tonn T., Schwabe D., Klingemann H.G., Becker S., Esser R., Koehl U., Suttorp M., Seifried E., Ottmann O.G., Bug G. (2013). Treatment of patients with advanced cancer with the natural killer cell line NK-92. Cytotherapy.

[148] Arai S., Meagher R., Swearingen M., Myint H., Rich E., Martinson J., Klingemann H. (2008). Infusion of the allogeneic cell line NK-92 in patients with advanced renal cell cancer or melanoma: A phase I trial. Cytotherapy.

[149] Romanski A., Uherek C., Bug G., Seifried E., Klingemann H., Wels W.S., Ottmann O.G., Tonn T. (2016). CD19-CAR engineered NK-92 cells are sufficient to overcome NK cell resistance in B-cell malignancies. J. Cell. Mol. Med..

[150] Sarvaria A., Jawdat D., Madrigal J.A., Saudemont A. (2017). Umbilical cord blood natural killer cells, their characteristics, and potential clinical applications. Front. Immunol..

[151] Shah N., Martin-Antonio B., Yang H., Ku S., Lee D.A., Cooper L.J.N., Decker W.K., Li S., Robinson S.N., Sekine T. (2013). Antigen Presenting Cell-Mediated Expansion of Human Umbilical Cord Blood Yields Log-Scale Expansion of Natural Killer Cells with Anti-Myeloma Activity. PLoS ONE.

[152] Ayello J., Hochberg J., Flower A., Chu Y., Baxi L.V., Quish W., van de Ven C., Cairo M.S. (2017). Genetically re-engineered K562 cells significantly expand and functionally activate cord blood natural killer cells: Potential for adoptive cellular immunotherapy. Exp. Hematol..

[153] Liu E., Marin D., Banerjee P., Macapinlac H.A., Thompson P., Basar R., Kerbauy L.N., Overman B., Thall P., Kaplan M. (2020). Use of CAR-Transduced Natural Killer Cells in CD19-Positive Lymphoid Tumors. N. Engl. J. Med..

[154] Ni J., Miller M., Stojanovic A., Garbi N., Cerwenka A. (2012). Sustained effector function of IL-12/15/18-preactivated NK cells against established tumors. J. Exp. Med..

[155] Romee R., Schneider S.E., Leong J.W., Chase J.M., Keppel C.R., Sullivan R.P., Cooper M.A., Fehniger T.A. (2012). Cytokine activation induces human memory-like NK cells. Blood.

[156] Rosario M., Romee R., Schneider S.E., Leong J.W., Sullivan R.P., Fehniger T.A. (2014). Human Cytokine-Induced Memory-like (CIML) NK Cells Are Active Against Myeloid Leukemia in Vitro and in Vivo. Blood.

[157] Romee R., Rosario M., Berrien-Elliott M.M., Wagner J.A., Jewell B.A., Schappe T., Leong J.W., Abdel-Latif S., Schneider S.E., Willey S. (2016). Cytokine-induced memory-like natural killer cells exhibit enhanced responses against myeloid leukemia. Sci. Transl. Med..

[158] Srivastava S., Pelloso D., Feng H., Voiles L., Lewis D., Haskova Z., Whitacre M., Trulli S., Chen Y.J., Toso J. (2013). Effects of interleukin-18 on natural killer cells: Costimulation of activation through Fc receptors for immunoglobulin. Cancer Immunol. Immunother..

[159] Floros T., Tarhini A.A. (2015). Anticancer Cytokines: Biology and Clinical Effects of Interferon-α2, Interleukin (IL)-2, IL-15, IL-21, and IL-12. Semin. Oncol..

[160] Davis Z.B., Felices M., Verneris M.R., Miller J.S. (2015). Natural killer cell adoptive transfer therapy: Exploiting the first line of defense against cancer. Cancer J..

[161] Ito S., Bollard C.M., Carlsten M., Melenhorst J.J., Biancotto A., Wang E., Chen J., Kotliarov Y., Cheung F., Xie Z. (2014). Ultra-low dose interleukin-2 promotes immune-modulating function of regulatory t cells and natural killer cells in healthy volunteers. Mol. Ther..

[162] Levin A.M., Bates D.L., Ring A.M., Krieg C., Lin J.T., Su L., Moraga I., Raeber M.E., Bowman G.R., Novick P. (2012). Exploiting a natural conformational switch to engineer an interleukin-2 “superkine”. Nature.

[163] Sim G.C., Liu C., Wang E., Liu H., Creasy C., Dai Z., Overwijk W.W., Roszik J., Marincola F., Hwu P. (2016). IL2 variant circumvents ICOS+ regulatory T-cell expansion and promotes NK cell activation. Cancer Immunol. Res..

[164] Carmenate T., Pacios A., Enamorado M., Moreno E., Garcia-Martínez K., Fuente D., León K. (2013). Human IL-2 Mutein with Higher Antitumor Efficacy Than Wild Type IL-2. J. Immunol..

[165] Waldmann T.A. (2006). The biology of interleukin-2 and interleukin-15: Implications for cancer therapy and vaccine design. Nat. Rev. Immunol..

[166] Rubinstein M.P., Kovar M., Purton J.F., Cho J.H., Boyman O., Surh C.D., Sprent J. (2006). Converting IL-15 to a superagonist by binding to soluble IL-15Rα. Proc. Natl. Acad. Sci. USA.

[167] Conlon K.C., Lugli E., Welles H.C., Rosenberg S.A., Fojo A.T., Morris J.C., Fleisher T.A., Dubois S.P., Perera L.P., Stewart D.M. (2015). Redistribution, hyperproliferation, activation of natural killer cells and CD8 T cells, and cytokine production during first-in-human clinical trial of recombinant human interleukin-15 in patients with cancer. J. Clin. Oncol..

[168] Romee R., Cooley S., Berrien-Elliott M.M., Westervelt P., Verneris M.R., Wagner J.E., Weisdorf D.J., Blazar B.R., Ustun C., DeFor T.E. (2018). First-in-human phase 1 clinical study of the IL-15 superagonist complex ALT-803 to treat relapse after transplantation. Blood.

[169] Denman C.J., Senyukov V.V., Somanchi S.S., Phatarpekar P.V., Kopp L.M., Johnson J.L., Singh H., Hurton L., Maiti S.N., Huls M.H. (2012). Membrane-bound IL-21 promotes sustained Ex Vivo proliferation of human natural killer cells. PLoS ONE.

[170] Ciurea S.O., Schafer J.R., Bassett R., Denman C.J., Cao K., Willis D., Rondon G., Chen J., Soebbing D., Kaur I. (2017). Phase 1 clinical trial using mbIL21 ex vivo-expanded donor-derived NK cells after haploidentical transplantation. Blood.

[171] Lim S.H., Beers S.A., French R.R., Johnson P.W.M., Glennie M.J., Cragg M.S. (2010). Anti-CD20 monoclonal antibodies: Historical and future perspectives. Haematologica.

[172] Labrijn A.F., Janmaat M.L., Reichert J.M., Parren P.W.H.I. (2019). Bispecific antibodies: A mechanistic review of the pipeline. Nat. Rev. Drug Discov..

[173] Koch J., Tesar M. (2017). Recombinant Antibodies to Arm Cytotoxic Lymphocytes in Cancer Immunotherapy. Transfus. Med. Hemotherapy.

[174] Gleason M.K., Verneris M.R., Todhunter D.A., Zhang B., McCullar V., Zhou S.X., Panoskaltsis-Mortari A., Weiner L.M., Vallera D.A., Miller J.S. (2012). Bispecific and trispecific killer cell engagers directly activate human NK cells through CD16 signaling and induce cytotoxicity and cytokine production. Mol. Cancer Ther..

[175] Chan W.K., Kang S., Youssef Y., Glankler E.N., Barrett E.R., Carter A.M., Ahmed E.H., Prasad A., Chen L., Zhang J. (2018). A CS1-NKG2D bispecific antibody collectivel activates cytolytic immune cells against multiple myeloma. Cancer Immunol. Res..

[176] Jelinek T., Mihalyova J., Kascak M., Duras J., Hajek R. (2017). PD-1/PD-L1 inhibitors in haematological malignancies: Update 2017. Immunology.

[177] Romagné F., André P., Spee P., Zahn S., Anfossi N., Gauthier L., Capanni M., Ruggeri L., Benson D.M., Blaser B.W. (2009). Preclinical characterization of 1-7F9, a novel human anti-KIR receptor therapeutic antibody that augments natural killer-mediated killing of tumor cells. Blood.

[178] Vey N., Bourhis J.H., Boissel N., Bordessoule D., Prebet T., Charbonnier A., Etienne A., Andre P., Romagne F., Benson D. (2012). Aphase 1 trial of the anti-inhibitory KIR mAb IPH2101 for AML in complete remission. Blood.

[179] McWilliams E.M., Mele J.M., Cheney C., Timmerman E.A., Fiazuddin F., Strattan E.J., Mo X., Byrd J.C., Muthusamy N., Awan F.T. (2016). Therapeutic CD94/NKG2A blockade improves natural killer cell dysfunction in chronic lymphocytic leukemia. Oncoimmunology.

[180] Wennerberg E., Kremer V., Childs R., Lundqvist A. (2015). CXCL10-induced migration of adoptively transferred human natural killer cells toward solid tumors causes regression of tumor growth in vivo. Cancer Immunol. Immunother..

[181] Vitale M., Cantoni C., Pietra G., Mingari M.C., Moretta L. (2014). Effect of tumor cells and tumor microenvironment on NK-cell function. Eur. J. Immunol..

[182] Yang Y., Lim O., Kim T.M., Ahn Y.O., Choi H., Chung H., Min B., Her J.H., Cho S.Y., Keam B. (2016). Phase I study of random healthy donor-derived allogeneic natural killer cell therapy in patients with malignant lymphoma or advanced solid tumors. Cancer Immunol. Res..

[183] Chambers A.M., Lupo K.B., Matosevic S. (2018). Tumor microenvironment-induced immunometabolic reprogramming of natural killer cells. Front. Immunol..

[184] Hasmim M., Messai Y., Ziani L., Thiery J., Bouhris J.H., Noman M.Z., Chouaib S. (2015). Critical role of tumor microenvironment in shaping NK cell functions: Implication of hypoxic stress. Front. Immunol..

[185] Mao Y., Sarhan D., Steven A., Seliger B., Kiessling R., Lundqvist A. (2014). Inhibition of tumor-derived prostaglandin-E2 blocks the induction of myeloid-derived suppressor cells and recovers natural killer cell activity. Clin. Cancer Res..

[186] Kim J., Kim J.S., Lee H.K., Kim H.S., Park E.J., Choi J.E., Choi Y.J., Shin B.R., Kim E.Y., Hong J.T. (2018). CXCR3-deficient natural killer cells fail to migrate to B16F10 melanoma cells. Int. Immunopharmacol..

[187] Kremer V., Ligtenberg M.A., Zendehdel R., Seitz C., Duivenvoorden A., Wennerberg E., Colón E., Scherman-Plogell A.-H., Lundqvist A. (2017). Genetic engineering of human NK cells to express CXCR2 improves migration to renal cell carcinoma. J. Immunother. Cancer.

[188] Parkhurst M.R., Riley J.P., Dudley M.E., Rosenberg S.A. (2011). Adoptive transfer of autologous natural killer cells leads to high levels of circulating natural killer cells but does not mediate tumor regression. Clin. Cancer Res..

[189] Talleur A.C., Triplett B.M., Federico S., Mamcarz E., Janssen W., Wu J., Shook D., Leung W., Furman W.L. (2017). Consolidation Therapy for Newly Diagnosed Pediatric Patients with High-Risk Neuroblastoma Using Busulfan/Melphalan, Autologous Hematopoietic Cell Transplantation, Anti-GD2 Antibody, Granulocyte-Macrophage Colony–Stimulating Factor, Interleukin-2, and Hapl. Biol. Blood Marrow Transplant..

[190] Chen X., Han J., Chu J., Zhang L., Zhang J., Chen C., Chen L., Wang Y., Wang H., Yi L. (2016). A combinational therapy of EGFR-CAR NK cells and oncolytic herpes simplex virus 1 for breast cancer brain metastases. Oncotarget.

[191] Schönfeld K., Sahm C., Zhang C., Naundorf S., Brendel C., Odendahl M., Nowakowska P., Bönig H., Köhl U., Kloess S. (2015). Selective inhibition of tumor growth by clonal NK cells expressing an ErbB2/HER2-specific chimeric antigen receptor. Mol. Ther..

[192] Zhang C., Burger M.C., Jennewein L., Genßler S., Schönfeld K., Zeiner P., Hattingen E., Harter P.N., Mittelbronn M., Tonn T. (2016). ErbB2/HER2-Specific NK Cells for Targeted Therapy of Glioblastoma. J. Natl. Cancer Inst..

[193] Murakami T., Nakazawa T., Natsume A., Nishimura F., Nakamura M., Matsuda R., Omoto K., Tanaka Y., Shida Y., Park Y.S. (2018). Novel human NK cell line carrying CAR targeting EGFRvIII induces antitumor effects in glioblastoma cells. Anticancer Res..

[194] Esser R., Müller T., Stefes D., Kloess S., Seidel D., Gillies S.D., Aperlo-Iffland C., Huston J.S., Uherek C., Schönfeld K. (2012). NK cells engineered to express a GD 2-specific antigen receptor display built-in ADCC-like activity against tumour cells of neuroectodermal origin. J. Cell. Mol. Med..

[195] Sahm C., Schönfeld K., Wels W.S. (2012). Expression of IL-15 in NK cells results in rapid enrichment and selective cytotoxicity of gene-modiWed eVectors that carry a tumor-speciWc antigen receptor. Cancer Immunol. Immunother..

[196] Li Y., Hermanson D.L., Moriarity B.S., Kaufman D.S. (2018). Human iPSC-Derived Natural Killer Cells Engineered with Chimeric Antigen Receptors Enhance Anti-tumor Activity. Cell Stem Cell.

[197] Guo C., Wang X., Zhang H., Zhi L., Lv T., Li M., Lu C., Zhu W. (2019). Structure-based rational design of a novel chimeric PD1-NKG2D receptor for natural killer cells. Mol. Immunol..

[198] Töpfer K., Cartellieri M., Michen S., Wiedemuth R., Müller N., Lindemann D., Bachmann M., Füssel M., Schackert G., Temme A. (2015). DAP12-Based Activating Chimeric Antigen Receptor for NK Cell Tumor Immunotherapy. J. Immunol..

[199] Xiao L., Cen D., Gan H., Sun Y., Huang N., Xiong H., Jin Q., Su L., Liu X., Wang K. (2019). Adoptive Transfer of NKG2D CAR mRNA-Engineered Natural Killer Cells in Colorectal Cancer Patients. Mol. Ther..

[200] Mensali N., Dillard P., Hebeisen M., Lorenz S., Theodossiou T., Myhre M.R., Fåne A., Gaudernack G., Kvalheim G., Myklebust J.H. (2019). NK cells specifically TCR-dressed to kill cancer cells. EBioMedicine.

[201] Parlar A., Sayitoglu E.C., Ozkazanc D., Georgoudaki A.M., Pamukcu C., Aras M., Josey B.J., Chrobok M., Branecki S., Zahedimaram P. (2019). Engineering antigen-specific NK cell lines against the melanoma-associated antigen tyrosinase via TCR gene transfer. Eur. J. Immunol..

[202] Jochems C., Hodge J.W., Fantini M., Fujii R., Morillon Y.M., Greiner J.W., Padget M.R., Tritsch S.R., Tsang K.Y., Campbell K.S. (2016). An NK cell line (haNK) expressing high levels of granzyme and engineered to express the high affinity CD16 allele. Oncotarget.

[203] Knorr D.A., Ni Z., Hermanson D., Hexum M.K., Bendzick L., Cooper L.J.N., Lee D.A., Kaufman D.S. (2013). Clinical-Scale Derivation of Natural Killer Cells From Human Pluripotent Stem Cells for Cancer Therapy. Stem Cells Transl. Med..

[204] Hermanson D.L., Bendzick L., Pribyl L., McCullar V., Vogel R.I., Miller J.S., Geller M.A., Kaufman D.S. (2016). Induced Pluripotent Stem Cell-Derived Natural Killer Cells for Treatment of Ovarian Cancer. Stem Cells.

[205] Rosenberg S.A. (2014). IL-2: The First Effective Immunotherapy for Human Cancer. J. Immunol..

[206] Han K.P., Zhu X., Liu B., Jeng E., Kong L., Yovandich J.L., Vyas V.V., Marcus W.D., Chavaillaz P.A., Romero C.A. (2011). IL-15:IL-15 receptor alpha superagonist complex: High-level co-expression in recombinant mammalian cells, purification and characterization. Cytokine.

[207] Thompson J.A., Curti B.D., Redman B.G., Bhatia S., Weber J.S., Agarwala S.S., Sievers E.L., Hughes S.D., Devries T.A., Hausman D.F. (2008). Phase I study of recombinant interleukin-21 in patients with metastatic melanoma and renal cell carcinoma. J. Clin. Oncol..

[208] Petrella T.M., Tozer R., Belanger K., Savage K.J., Wong R., Smylie M., Kamel-Reid S., Tron V., Chen B.E., Hunder N.N. (2012). Interleukin-21 has activity in patients with metastatic melanoma: A phase II study. J. Clin. Oncol..

[209] Grünwald V., Desar I.M.E., Haanen J., Fiedler W., Mouritzen U., Olsen M.W.B., Van Herpen C.M.L. (2011). A Phase i study of recombinant human interleukin-21 (rIL-21) in combination with sunitinib in patients with metastatic renal cell carcinoma (RCC). Acta Oncol..

[210] Rhode P.R., Egan J.O., Xu W., Hong H., Webb G.M., Chen X., Liu B., Zhu X., Wen J., You L. (2016). Comparison of the superagonist complex, ALT-803, to IL15 as cancer immunotherapeutics in animal models. Cancer Immunol. Res..

[211] Tang F., Zhao L., Jiang Y., Ba D., Cui L., He W. (2008). Activity of recombinant human interleukin-15 against tumor recurrence and metastasis in mice. Cell. Mol. Immunol..

[212] Pérez-Martínez A., Fernández L., Valentín J., Martínez-Romera I., Corral M.D., Ramírez M., Abad L., Santamaría S., González-Vicent M., Sirvent S. (2015). A phase I/II trial of interleukin-15-stimulated natural killer cell infusion after haplo-identical stem cell transplantation for pediatric refractory solid tumors. Cytotherapy.

[213] Wrangle J.M., Velcheti V., Patel M.R., Garrett-Mayer E., Hill E.G., Ravenel J.G., Miller J.S., Farhad M., Anderton K., Lindsey K. (2018). ALT-803, an IL-15 superagonist, in combination with nivolumab in patients with metastatic non-small cell lung cancer: A non-randomised, open-label, phase 1b trial. Lancet Oncol..

[214] Moroz A., Eppolito C., Li Q., Tao J., Clegg C.H., Shrikant P.A. (2004). IL-21 Enhances and Sustains CD8 + T Cell Responses to Achieve Durable Tumor Immunity: Comparative Evaluation of IL-2, IL-15, and IL-21. J. Immunol..

[215] Parrish-Novak J., Dillon S.R., Nelson A., Hammond A., Sprecher C., Gross J.A., Johnston J., Madden K., Xu W., West J. (2000). Interleukin 21 and its receptor are involved in NK cell expansion and regulation of lymphocyte function. Nature.

[216] Wang G., Tschoi M., Spolski R., Lou Y., Ozaki K., Feng C., Kim G., Leonard W., Hwu P. (2003). In vivo antitumor activity of interleukin 21 mediated by natural killer cells. Cancer Res..

[217] Di Carlo E., Comes A., Orengo A.M., Rosso O., Meazza R., Musiani P., Colombo M.P., Ferrini S. (2004). IL-21 Induces Tumor Rejection by Specific CTL and IFN-γ-Dependent CXC Chemokines in Syngeneic Mice. J. Immunol..

[218] Stolfi C., Pallone F., Macdonald T.T., Monteleone G. (2012). Interleukin-21 in cancer immunotherapy: Friend or foe?. Oncoimmunology.

[219] Umaña P., Jean-Mairet J., Moudry R., Amstutz H., Bailey J.E. (1999). Engineered glycoforms of an antineuroblastoma IgG1 with optimized antibody-dependent cellular cytotoxic activity. Nat. Biotechnol..

[220] Lazar G.A., Dang W., Karki S., Vafa O., Peng J.S., Hyun L., Chan C., Chung H.S., Eivazi A., Yoder S.C. (2006). Engineered antibody Fc variants with enhanced effector function. Proc. Natl. Acad. Sci. USA.

[221] Xie Z., Shi M., Feng J., Yu M., Sun Y., Shen B., Guo N. (2003). A trivalent anti-erbB2/anti-CD16 bispecific antibody retargeting NK cells against human breast cancer cells. Biochem. Biophys. Res. Commun..

[222] Asano R., Nakayama M., Kawaguchi H., Kubota T., Nakanishi T., Umetsu M., Hayashi H., Katayose Y., Unno M., Kudo T. (2012). Construction and humanization of a functional bispecific EGFR × CD16 diabody using a refolding system. FEBS J..

[223] Schmohl J.U., Felices M., Taras E., Miller J.S., Vallera D.A. (2016). Enhanced ADCC and NK cell activation of an anticarcinoma bispecific antibody by genetic insertion of a modified IL-15 cross-linker. Mol. Ther..

[224] Bellone S., Black J., English D.P., Schwab C.L., Lopez S., Cocco E., Bonazzoli E., Predolini F., Ferrari F., Ratner E. (2016). Solitomab, an EpCAM/CD3 bispecific antibody construct (BiTE), is highly active against primary uterine serous papillary carcinoma cell lines in vitro. Am. J. Obstet. Gynecol..

[225] Xu F., Sunderland A., Zhou Y., Schulick R.D., Edil B.H., Zhu Y. (2017). Blockade of CD112R and TIGIT signaling sensitizes human natural killer cell functions. Cancer Immunol. Immunother..

[226] Blake S.J., Stannard K., Liu J., Allen S., Yong M.C.R., Mittal D., Aguilera A.R., Miles J.J., Lutzky V.P., de Andrade L.F. (2016). Suppression of metastases using a new lymphocyte checkpoint target for cancer immunotherapy. Cancer Discov..

[227] Da Silva I.P., Gallois A., Jimenez-Baranda S., Khan S., Anderson A.C., Kuchroo V.K., Osman I., Bhardwaj N. (2014). Reversal of NK-cell exhaustion in advanced melanoma by Tim-3 blockade. Cancer Immunol. Res..

[228] Lu S., Zhang J., Liu D., Li G., Staveley-O’Carroll K.F., Li Z., Wu J.D. (2015). Nonblocking monoclonal antibody targeting soluble MIC revamps endogenous innate and adaptive antitumor responses and eliminates primary and metastatic tumors. Clin. Cancer Res..

[229] Zhang J., Liu D., Li G., Staveley-O’Carroll K.F., Graff J.N., Li Z., Wu J.D. (2017). Antibody-mediated neutralization of soluble MIC significantly enhances CTLA4 blockade therapy. Sci. Adv..

[230] Kamiya T., Seow S.V., Wong D., Robinson M., Campana D. (2019). Blocking expression of inhibitory receptor NKG2A overcomes tumor resistance to NK cells. J. Clin. Investig..

[231] Zaghi E., Calvi M., Marcenaro E., Mavilio D., Di Vito C. (2019). Targeting NKG2A to elucidate natural killer cell ontogenesis and to develop novel immune-therapeutic strategies in cancer therapy. J. Leukoc. Biol..

[232] Sarhan D., Wennerberg E., D’Arcy P., Gurajada D., Linder S., Lundqvist A. (2013). A novel inhibitor of proteasome deubiquitinating activity renders tumor cells sensitive to TRAIL-mediated apoptosis by natural killer cells and T cells. Cancer Immunol. Immunother..

[233] Hallett W.H.D., Ames E., Motarjemi M., Barao I., Shanker A., Tamang D.L., Sayers T.J., Hudig D., Murphy W.J. (2008). Sensitization of Tumor Cells to NK Cell-Mediated Killing by Proteasome Inhibition. J. Immunol..

[234] Wennerberg E., Sarhan D., Carlsten M., Kaminskyy V.O., D’Arcy P., Zhivotovsky B., Childs R., Lundqvist A. (2013). Doxorubicin sensitizes human tumor cells to NK cell- and T-cell-mediated killing by augmented TRAIL receptor signaling. Int. J. Cancer.

[235] Cifaldi L., Locatelli F., Marasco E., Moretta L., Pistoia V. (2017). Boosting Natural Killer Cell-Based Immunotherapy with Anticancer Drugs: A Perspective. Trends Mol. Med..

[236] Guillerey C., Huntington N.D., Smyth M.J. (2016). Targeting natural killer cells in cancer immunotherapy. Nat. Immunol..

